# *Bacillus vallismortis* LRB-5: a promising biocontrol agent for mitigating apple replant disease through pathogen suppression and growth promotion

**DOI:** 10.1007/s44154-025-00246-5

**Published:** 2025-08-25

**Authors:** Yanan Duan, Ziqing Ma, Yiwei Jia, Hengtong Xing, Zhiquan Mao, Ke Mao, Zhijun Zhang, Chao Li, Fengwang Ma

**Affiliations:** 1 State Key Laboratory for Crop Stress Resistance and High-Efficiency Production/Shaanxi Key Laboratory of Apple, College of Horticulture, Northwest A&F University, Yangling, 712100 Shaanxi China; 2 College of Horticulture Science and Engineering, Shandong Agricultural University, National Key Laboratory of Crop Biology, Taian, 271018 Shangdong China

**Keywords:** Apple replant disease, *Fusarium* spp., Biological control, *Bacillus vallismortis*

## Abstract

Apple replant disease (ARD) poses a serious threat to apple cultivation, primarily caused by the accumulation of *Fusarium* species. *Bacillus* species have demonstrated significant potential as microbial agents, with capabilities in promoting plant growth, suppressing soil-borne pathogens, and improving soil quality. Here in this study, strain LRB-5 was isolated from a healthy apple root system and identified as *Bacillus vallismortis* based on physiological and biochemical characterization and molecular sequencing analysis. It exhibited broad-spectrum antifungal activity against various *Fusarium* species, including *F. oxysporum*, *F. moniliforme*, *F. proliferatum*, and *F. solani*, with inhibition rates exceeding 65%. LRB-5 extracellular metabolites significantly inhibited *Fusarium* mycelial growth and spore germination. Greenhouse experiments demonstrated that LRB-5 reduced ARD disease severity by more than 50%. The volatile organic compounds produced by LRB-5 exhibited both antimicrobial activity and growth-promoting properties. Further assays revealed LRB-5 can secrete various cell wall-degrading enzymes and possesses plant growth-promoting capabilities. Pot experiments showed LRB-5 had excellent colonization ability in the rhizosphere of *Malus hupehensis* Rehd. seedlings, significantly increasing seedling biomass, soil bacterial and actinomycete populations, and the activity of root protective enzymes. Moreover, LRB-5 significantly enhanced the activity of soil enzymes while reducing the contents of phlorizin, benzoic acid, and *p*-hydroxybenzoic acid in the rhizosphere soil. Terminal restriction fragment length polymorphism and quantitative real-time PCR analyses revealed that LRB-5 improved bacterial carbon utilization, increased microbial diversity indices, reduced the abundance of *Fusarium* spp., and altered the structure of soil microbial communities. Collectively, these rusults suggest that LRB-5 effectively alleviated ARD by protecting apple roots from *Fusarium* infection and phenolic acid toxicity, optimizing soil microbial communities, and promoting plant growth. Future research should explore the combined application of LRB-5 with other control measures, thereby promoting its practical implementation.

**Supplementary Information:**

The online version contains supplementary material available at 10.1007/s44154-025-00246-5.

## Introduction

The apple industry is a crucial part of China’s agriculture sector, playing a significant role in industrial restructuring, increasing farmers'income, and generating foreign exchange through exports (Liang et al. 2022). However, owing to limited land resources, over 70% of orchards face the issue of aging trees and an urgent need for their replacement and renewal (Zhang et al. 2021). Replanting in the original locations of old orchards is inevitable, and apple replant disease (ARD) is common (Wang et al. 2022). ARD can cause newly planted apple saplings to exhibit weak growth, limited branch development, inadequate root systems, root rot, delayed fruiting, and low yields (Wang et al. 2024). Generally, it can reduce yields by 20–50%, and in severe cases, lead to complete crop failure (Xiang et al. 2022). This results in substantial losses for fruit farmers and severely hinders the sustainable development of the apple industry.

The occurrence of ARD is the result of the combined action of multiple factors (Duan et al. 2022c), with soil microbial factors being the primary cause. The roots and residues of previous apple trees can introduce many soil-borne pathogens, including fungi (*Rhizoctonia solani*, *Ilyonectria*, *Fusarium*), oomycetes (*Pythium* and *Phytophthora*), and nematodes (*Pratylenchus* spp.). These pathogens can invade the roots of newly planted apple trees (Duan et al. 2022c; Hewavitharana and Mazzola 2016; Kelderer et al. 2012; Tewoldemedhin et al. 2011; Tilston et al. 2018; Van Schoor et al. 2009; Wang et al. 2018). Previous studies have confirmed that *Fusarium* spp. were key pathogens causing ARD in major apple-producing regions including China (Duan et al. 2022c; Xiang et al. 2021), South Africa (Van Schoor et al. 2009), and Italy (Kelderer et al. 2012). In China, *F. solani*, *F. proliferatum*, *F. moniliforme*/*verticillioides*, and *F. oxysporum* have demonstrated strong pathogenicity against apple rootstocks, with their populations showing significant increases in replanted soils (Duan et al. 2022c; Sheng et al. 2020; Wang et al. 2018; Zhang et al. 2021). Duan et al. (2022c) identified *Fusarium proliferatum* f.sp. *malus domestica* MR5 (*Fpmd* MR5) as a highly pathogenic strain causing ARD in China. At the same time, the number of beneficial microorganisms (such as some actinomycetes and *Bacillus*, which can promote plant growth and inhibit pathogens) decreases (Balbín-Suárez et al. 2021). The microbial community structure can thus become unbalanced, leading to ineffective suppression of harmful microorganisms. This results in root rot and growth retardation, ultimately affecting the growth and development of the above-ground parts. ARD can also cause changes in the soil’s physical and chemical properties, such as nutrient imbalance (especially the excessive depletion of trace elements like iron, zinc, and manganese). Additionally, the soil can become compacted, with poor aeration and drainage, and undergo acidification, which negatively impacts root respiration and nutrient absorption (Cesarano et al. 2017; Wang et al. 2014). In addition, metabolic products secreted by apple tree roots, such as cinnamic acid, *p*-hydroxybenzoic acid, phthalic acid, phlorizin, and benzoic acid, accumulate in the soil, producing toxic substances that inhibit the growth and development of newly planted apple tree roots (Bai et al. 2019; Gao et al. 2010; Yin et al. 2016, 2017; Wang et al. 2014).

Currently, the main methods for controlling ARD include soil improvement (such as deep plowing, biochar addition, rational fertilization, pH adjustment, and chemical fumigation), planting resistant varieties, and cultivation management (such as crop rotation, intercropping, and mixed planting) (He et al. 2023; Liu et al. 2024; Mechler 2024). However, these methods have numerous drawbacks, including high risks of failure, environmental pollution, time consumption, high costs, short-term inefficacy, and operational complexity, leading to their gradual abandonment (Raymaekers et al. 2020; Yan et al. 2021). In recent years, biological control measures involving the addition of beneficial microbes (such as *Bacillus*, *Pseudomonas*, *Trichoderma*, and *Streptomyces*) have gained increasing attention from researchers owing to their environmental friendliness and efficiency and have thus been widely adopted by fruit growers (Liu et al. 2019; Raza et al. 2016; Xu et al. 2020). Among these beneficial microbes, biological agents primarily composed of *Bacillus* spp. have shown significant efficacy in controlling agricultural pests and improving crop growth environments (Chen et al. 2020; Etesami et al. 2023). *Bacillus* can colonize the rhizosphere and roots of plants, thus establishing a symbiotic relationship. Moreover, *Bacillus* spp. have been reported to occupy ecological niches through competition, produce antimicrobial substances (such as antibiotics, bacteriocins, and extracellular hydrolases), induce plant resistance to enhance immunity (e.g., by promoting nutrient solubilization and producing plant hormones), and improve soil environments to facilitate nutrient transformation, thereby effectively controlling soil-borne diseases (Fan et al. 2023; Pradhan et al. 2023; Sarangi and Ramakrishnan 2023; Wu et al. 2019). Biological agents offer key advantages in crop management, such as being environmentally friendly, non-toxic, and residue-free, having a broad control spectrum, simultaneously managing multiple pests and diseases, promoting crop growth, improving yield and quality, and providing long-lasting efficacy with reduced application frequency. Consequently, they are widely used in the cultivation of various crops, including staple grain crops (such as rice, wheat, and corn), cash crops (such as vegetables, fruits, flowers, and tea), cotton, tobacco, and medicinal plants (Jiao et al., 2020; Marković et al. 2023; Pradhan et al. 2023; Thanh et al. 2009; Thepbandit et al. 2023; Teli 2024; Xu et al. 2023). However, it is important to avoid mixing biological agents with fungicides and to store them in cool, dry, and ventilated conditions to prevent the inactivation of *Bacillus*. Notably, recent studies have shown that certain treatments including *Bacillus* exhibit significant antagonistic effects against common pathogens associated with ARD. For example, adding a compound microbial agent containing 2.6 × 10⁹ CFU·g^−1^ of *Bacillus* to the soil significantly increased the biomass of apple plants affected by ARD (Geng et al. 2022). Nevertheless, *Bacillus* resources with biocontrol effects against the four common *Fusarium* species related to ARD remain relatively scarce.

ARD caused by pathogenic *Fusarium* poses a great threat to the renewal and replacement of various apple varieties in China. Therefore, the development of effective biological control agents has become a crucial strategy for managing ARD. *Bacillus* spp. have shown great potential in promoting plant growth and resisting pathogen infections, but research on their specific efficacy against *Fusarium* spp. associated with ARD remains limited. In this study, *Bacillus vallismortis* LRB-5, a strain with broad-spectrum antifungal activity, was isolated from the root system of a healthy apple tree in a replanted orchard. The objectives of the present study included the following: (1) explore the mechanism by which LRB-5 inhibits the activity of plant pathogens; (2) evaluate the control effect of LRB-5 against *Fusarium* spp.; (3) investigate the role of LRB-5 in controlling ARD, thereby laying an empirical foundation for the biological control of ARD.

## Results

### The rhizosphere soil environment and antagonistic bacteria

In the replanted orchard, the available phosphorus content was 159.12 mg·kg⁻^1^, available potassium content was 143.89 mg·kg⁻^1^, organic matter content was 1.83%, soil bulk density was 1.15 g·cm⁻^3^, available nitrogen content was 25.66 mg·kg⁻^1^, pH was 6.85, soil moisture was 13.11%, and the soil texture was sandy loam in the rhizosphere soil of healthy trees (Table S3). ARD severity was calculated as the ratio of dry biomass produced in sterilized and unsterilized soil from the same orchard, and the soils were accordingly classified into severe, moderate, and low severity categories. The old apple orchard soil was determined to exhibit severe ARD (Table S3). The proportion of strains showing biocontrol potential varied among different sites, ranging from 14.29% to 41.67%. LRB-5 was isolated from the root system of a healthy apple tree in a replanted orchard in Longkou City, Shandong Province, China (Table S4). It exhibited broad-spectrum antagonistic activity against fungal pathogens, and its inhibitory effect was significantly higher than that of the other isolates tested (Table 1, Fig. S2). The inhibition rates of LRB-5 against *F. oxysporum* (D1), *A. alternata*, and *V. mali* were high, reaching 76.00%, 83.26%, and 82.30% on PDA, respectively. The inhibition rates against *R. solani*, *P. macrostoma*, *P. brasilianum*, *F. proliferatum*, *F. verticillioides*, and *F. solani* also exceeded 65%. Therefore, LRB-5 was selected for further evaluation of its potential to control ARD.

**Table 1:** Antifungal activity of LRB-5 against plant pathogens

Treatment	Colony diameter (cm)	Inhibition zone (mm)	Inhibition rate (%)^a^	Spore germination rate (%)^b^			
				CK1^c^	CK2	LRB-5	CFCF
*Fusarium proliferatum* (A1)	1.47 ± 0.02b	+ + +	67.33 ± 0.38e	96.77 ± 0.33a	95.96 ± 1.27a	40.98 ± 3.33c	35.46 ± 1.12a
*Fusarium verticillioides* (B1)	1.23 ± 0.01d	+ + +	72.67 ± 0.22c	97.52 ± 0.77a	96.12 ± 0.37a	46.43 ± 0.82b	28.23 ± 1.57b
*Fusarium solani* (C1)	1.18 ± 0.03d	+ + +	73.78 ± 0.59c	96.75 ± 0.33a	96.78 ± 0.61a	34.44 ± 3.84d	23.26 ± 1.51c
*Fusarium oxysporum* (D1)	1.08 ± 0.06e	+ + +	76.00 ± 1.24b	98.19 ± 0.27a	97.45 ± 1.10a	36.53 ± 0.85d	22.80 ± 1.85c
*Fusarium proliferatum *MR5 (A2)	1.47 ± 0.01b	+ + +	67.26 ± 0.13e	97.22 ± 1.54a	95.45 ± 0.84a	57.11 ± 0.98a	37.10 ± 2.51a
*Fusarium verticillioides *YR15 (B2)	1.54 ± 0.02b	+ + +	65.85 ± 0.47e	-	-	-	-
*Fusarium solani* Q61 (C2)	1.24 ± 0.03d	+ + +	72.30 ± 0.71c	-	-	-	-
*Fusarium oxysporum *HC131 (D2)	1.24 ± 0.03d	+ +	72.37 ± 0.71c	-	-	-	-
*Alternaria alternata*	0.75 ± 0.07f	+ + +	83.26 ± 1.48a	-	-	-	-
*Rhizoctonia solani*	1.37 ± 0.04c	+ +	69.63 ± 0.93d	-	-	-	-
*Valsa mali*	0.80 ± 0.02f	+ + +	82.30 ± 0.46a	-	-	-	-
*Aspergillus flavus*	2.55 ± 0.05a	+	43.33 ± 1.02f	-	-	-	-
*Albifimbria verrucaria*	0.50 ± 0.10g	+ + +	33.33 ± 2.22g	-	-	-	-
*Penicillium brasilianum*	1.34 ± 0.05c	+ +	70.07 ± 1.12d	-	-	-	-
*Phoma macrostoma* HC139	1.24 ± 0.03d	+ + +	72.37 ± 0.56c	-	-	-	-

–, No inhibition zone; +, weak inhibition with inhibition zone < 5 mm, growth of the fungus was stopped at the bacterial-streak line; + +, moderate inhibition with inhibition zone 5–10 mm; + + +, strong inhibition with inhibition zone > 10 mm. There were 3 replicated plates for each fungal-strain LRB-5 medium combination, and the experiment was repeated once. A zone of hyphal growth inhibition appeared around the bacterial colony. Values in columns followed by the same letter are not significantly different according to Duncan's test at *p* < 0.05. Values are mean ± standard deviation (*n* = 3). Column comparison: differences in spore germination rate between the control group and experimental groups; row comparison: inhibitory effects of LRB-5 against different plant pathogen

^a^Inhibition rate (PI) = (D − d)/D × 100%. D = diameter of pathogen growth in control plates (mm); d = diameter of pathogen growth in treated plates (mm)

^b^The germination rate was calculated as G/T × 100%, where G is the number of germinated spores, and T is the total number of spores observed. -: Not measured

^c^CK1: *Fusarium* spore suspension was mixed with sterile water at 1:1, CK2: *Fusarium* spore suspension was mixed with fermentation broth at 1:1 (*Bacillus* without antibacterial effect), LRB-5: *Fusarium* spore suspension was mixed with fermentation broth at 1:1, CFCF: *Fusarium* spore suspension was mixed with cell-free culture filtrate at 1:1

### Morphological observations

After LRB-5 had been cultured on LB agar for 24 h, each colony appeared light yellow, with a round or oval shape, and its surface was dull, dry, rough, and opaque. The edges were initially neat but later became irregular (Fig. S3A). LRB-5 was identified as a gram-positive, aerobic bacterium. The cells were straight, rod-shaped, measuring 0.5–1.0 × 1.0–2.5 μm (Fig. S3D–I), and the endospores were ovoid, measuring 0.5–0.7 × 1.0–2.3 μm, with mesophilic or subterminal positioning (Fig. S3B–C).

### Physiological and biochemical characteristics

Physiological and biochemical tests showed that LRB-5 was able to produce hydrogen peroxide, hydrolyze starch, reduce nitrate, and utilize glucose, sucrose, and citrate. The gelatin hydrolysis enzyme and contact enzyme were positive, whereas Voges–Proskauer reaction, indole enzyme, the malonate reaction, and methyl red reaction tests were negative (Table S5). The Biolog GEN III MicroStation System was used to identify LRB-5 based on its carbon utilization. The strain LRB-5 was able to utilize d-fructose, d-cellobiose, sucrose, α-d-glucose, gelatin, l-lactic acid, and citric acid as carbon sources. It was sensitive to pH 5, pH 6, 1% NaCl, 4% NaCl, 8% NaCl, 1% sodium lactate, and sodium butyrate (Table S6). According to *Bergey’s Manual of Systematic Bacteriology* (2nd edition) and the *Common Bacterial Identification Manual*, LRB-5 matched the physiological and biochemical properties of *Bacillus vallismortis*.

### Phylogenetic analysis

Approximately 558–937 bases were determined from *rpoB* and *gyrA*, 1455 bases from 16S rRNA, and 1085 bases from *gyrB*. Congruency analysis revealed no conflict among the 16S rRNA, *gyrA*, *gyrB*, and *rpoB* sequence datasets, and they were therefore combined. The combined four-loci sequence dataset included 38 ingroup taxa, with *Paenibacillus polymyxa* (BLB267) as the outgroup taxon. ML analysis of identities based on the four-gene sequence alignment between LRB-5 and the other strains revealed that its homology was highest with *B. vallismortis* (Fig. S4). In agreement with the analysis of physiological and biochemical characteristics, the sequence-based analysis indicated LRB-5 is *B. vallismortis*.

### Determination of antimicrobial activity

**Fig. 1:**
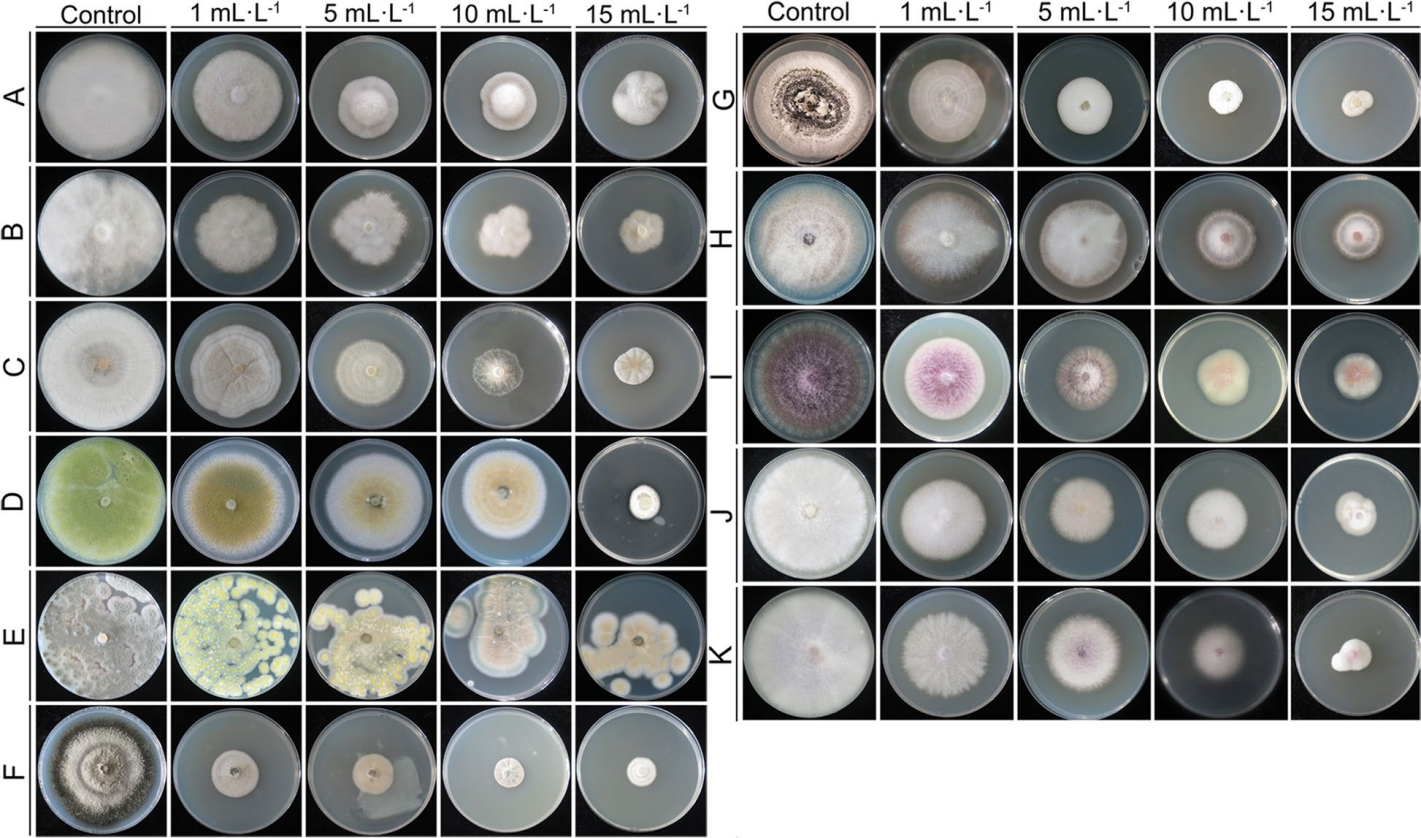
Effects of different concentrations of cell-free culture filtrate on the pathogen mycelial growth. The concentration of cell-free culture filtrate was 1, 5, 10, and 15 mL·L^−1^. **A** *Alternaria alternata*; **B** *Phytophthora cactorum*; **C** *Rhizoctonia solani*; **D** *Aspergillus flavus*; **E** *Penicillium brasilianum*; **F** *Phoma macrostoma*; **G** *Albifimbria verrucaria*; **H** *Fusarium proliferatum*; **I** *Fusarium verticillioides*; **J** *Fusarium solani*; **K** *Fusarium oxysporum*

Microscopic observations revealed that the control groups of *F. oxysporum* and *F. solani* exhibited uniform thickness, was slender, had few branches, full spores, a complete structure, and a healthy appearance (Fig. S5 A–D; Fig. S6 A–C). The mycelium treated with the LRB-5 cell-free culture filtrate was irregularly reticulated, uneven in thickness, shrunken, twisted, swollen, thinned, and fragmented, and had overflowing cell contents. The spore cell wall was also deformed (Fig. S5 E–H; Fig. S6D–H). Microscopy of *Fusarium* spore germination indicated that conidial germination was inhibited after treatment with LRB-5, but there were no changes in shape compared with the control. After 24 h of incubation, up to 95% of the conidia on the control slide (CK1), and the fermentation broth inoculated with non-biocontrol active *Bacillus* (CK2) produced germination tubes (Fig. S7, Table 1); however, approximately 60% of conidia did not germinate after fermentation broth and cell-free culture filtrate treatment (Fig. S7, Table 1). As the concentration of cell-free culture filtrate increased, the inhibitory effect on the growth of plant pathogens became more pronounced. For *F. oxysporum*, inhibition rates reached 60.06% at higher filtrate concentration, while *F. solani* showed 67.11% suppression under identical conditions (Fig. 1; Table S7).

### Volatile antimicrobial metabolites

LRB-5 showed significant inhibitory effects against plant pathogens in a bioassay without direct contact. The VOCs produced by LRB-5 significantly inhibited mycelial growth (Fig. S8). The total ion chromatogram (TIC) of the volatile metabolites from LRB-5 indicated that they were mainly concentrated between 10 and 40 min (Fig. S9). Based on this and the temperature program of the GC–MS, it can be inferred that the boiling point of most volatile metabolites from LRB-5 was no more than 250°C. The volatile metabolites were identified by GC–MS analysis followed by an NIST23 database search, and VOCs with a peak area percentage > 0.5, similarity index (SI) > 80, and retention index (RI) > 800 were identified. The results showed that LRB-5 produces 25 volatile substances, including 7 alcohols, 3 ketones, 5 alkanes, 2 organic acid esters, 1 aldehyde, 1 ether, 1 siloxane, 4 benzene derivatives, and 1 benzodiazepine. The relative contents of the above compounds are shown in Table 2. From among the 25 identified compounds, 9 pure compounds were purchased for individual testing of antimicrobial properties. The plant pathogens in the control group (sterile water) grew well. Nine pure compounds exhibited varying antimicrobial activities against all tested pathogens, all of which inhibited their growth, each with an inhibition rate of approximately 70% (Table S8, Fig. S10). Previously, *n*-pentadecanol, eicosane, 2-undecanone, dodecane, butylated hydroxytoluene, and 6-methyl-2-heptanol have been shown to have significant inhibitory effects on plant pathogens (Duan et al. 2022a).

**Table 2:** Volatile antimicrobial components of *Bacillus vallismortis* LRB-5 extract from different SPME fiber

Extraction head	Number	Retention time (min)	Are (%)	Ingredient name	Molecular formula	Molecular weight	Retention index	CAS number	Similarity Index (SI)
Blue	1	11.511	35.34	Toluene	C_7_H_8_	92.140	1035	108–88-3	97
	2	20.934	4.760	2,5-Dihydroxybenzaldehyde	C_7_H_6_O_3_	138.120	1578	1194–98-5	81
	3	35.862	2.88	Eicosane	C_20_H_42_	282.553	2000	112–95-8	91
	4	15.085	0.98	Styrene	C_8_H_8_	104.152	885	100–42-5	91
	5	17.134	1.45	5-methyl-2-heptanone	C_8_H_16_O	128.214	965	18217–12-4	81
	6	19.018	0.73	2-Ethyl-1-hexanol	C_8_H_18_O	130.230	1031	104–76-7	94
	7	23.895	0.69	Dodecane	C_12_H_26_	170.338	1200	112–40-3	88
	8	25.035	0.53	Nordiazepam, tert-butyldimethylsilyl derivative	C_21_H_25_ClN_2_OSi	384.000	2793	0–00-0	82
Red	1	10.091	16.97	Dimethylsiloxane	C_2_H_8_O_2_Si	92.1698	409	1066–42-8	98
	2	14.913	6.11	methoxyphenyl oxime	C_8_H_9_NO_2_	151.165	1332	0–00-0	90
	3	36.000	4.54	Hexanoic acid, 3,5,5-trimethyl-, pentyl ester	C_14_H_28_O_2_	228.000	1432	0–00-0	87
	4	16.347	4.11	4-[(2-Phenylethyl)carbamoyl]phenyl acetate, TMS	C_20_H_25_NO_3_Si	355.000	2301	0–00-0	87
	5	22.040	1.62	1,1,3,3,5,5,7,7,9,9-decamethylpentasiloxane	C_10_H_32_O_4_Si_5_	356.789	1118	995–83-5	87
	6	24.791	1.05	Benzimidazolemethanol, 5(or 6)-chloro-alpha-methyl-	C_15_H_25_ClN_2_OSi_2_	340.000	1718	0–00-0	94
Grey	1	28.369	1.00	1-(allyloxy)dodecane	C_15_H_30_O	226.400	1388	6145–80-8	82
	2	16.747	0.96	6-methyl-2-heptanol	C_8_H_18_O	130.230	951	4730–22-7	85
	3	28.365	0.89	11-Methyltricosane	C_24_H_50_	338.661	2343	27538–41-6	80
	4	28.890	0.84	1-Tetradecanol	C_14_H_30_O	214.392	1675	112–72-1	91
	5	26.521	0.83	2-Undecanone	C_11_H_22_O	170.295	1251	112–12-9	88
	6	33.705	0.75	Heptacosane	C_17_H_36_	380.742	2700	593–49-7	86
	7	28.892	0.75	n-Pentadecanol	C_15_H_30_O	228.418	1755	629–76-5	91
	8	22.965	0.74	6,10-dimethyl-undecan-2-one	C_13_H_26_O	198.349	1321	1604–34-8	84
	9	28.896	0.66	1-Hexadecanol	C_16_H_34_O	242.445	2384	36653–82-4	93
	10	31.437	0.60	Heneicosane	C_21_H_44_	296.580	2100	629–94-7	91
	11	32.233	0.59	Butylated Hydroxytoluene	C_15_H_24_O	220.355	1668	128–37-0	84

Blue: 65 μm PDMS/DVB, Red:100 μm PDMS, Grey: 50/30 μm PDMS/DVB/CAR

### PGP activities

LRB-5 possessed multiple PGP properties, such as phosphate and potassium solubilization; nitrogen fixation; indole-3-acetic acid (IAA) production (21.60 μg^.^mL^−1^; standard curve, *y* = 1.62406*x* + 0.00712, *R*^2^ = 0.99473); 1-aminocyclopropane-1-carboxylic acid (ACC) deaminase, ammonia, and amylase production; siderophore production; cell wall-degrading enzyme (cellulase, pectinase, β1,3-glucanase, and protease) production, and antifungal activity against phytopathogens (Fig. S11). An HPLC–MS/MS method was used to assay 31 free amino acids, 22 of which were detected (Table S9), including glutamine (Gln), asparagine (Asn), *S*-adenosyl methionine (*S*-Met), citrulline, tryptophan (Trp), l-kynurenine (Kyn), leucine (Leu), l-isoleucine (Ile), glycine (Gly), alanine (Ala), serine (Ser), proline (Pro), valine (Val), threonine (Thr), aspartic acid (Asp), lysine (Lys), glutamate (Glu), phenylalanine (Phe), arginine (Arg), tyrosine (Tyr), histidine (His), and methionine (Met).

### Growth promotion of *Arabidopsis thaliana* Col‐0 by VOCs

**Fig. 2:**
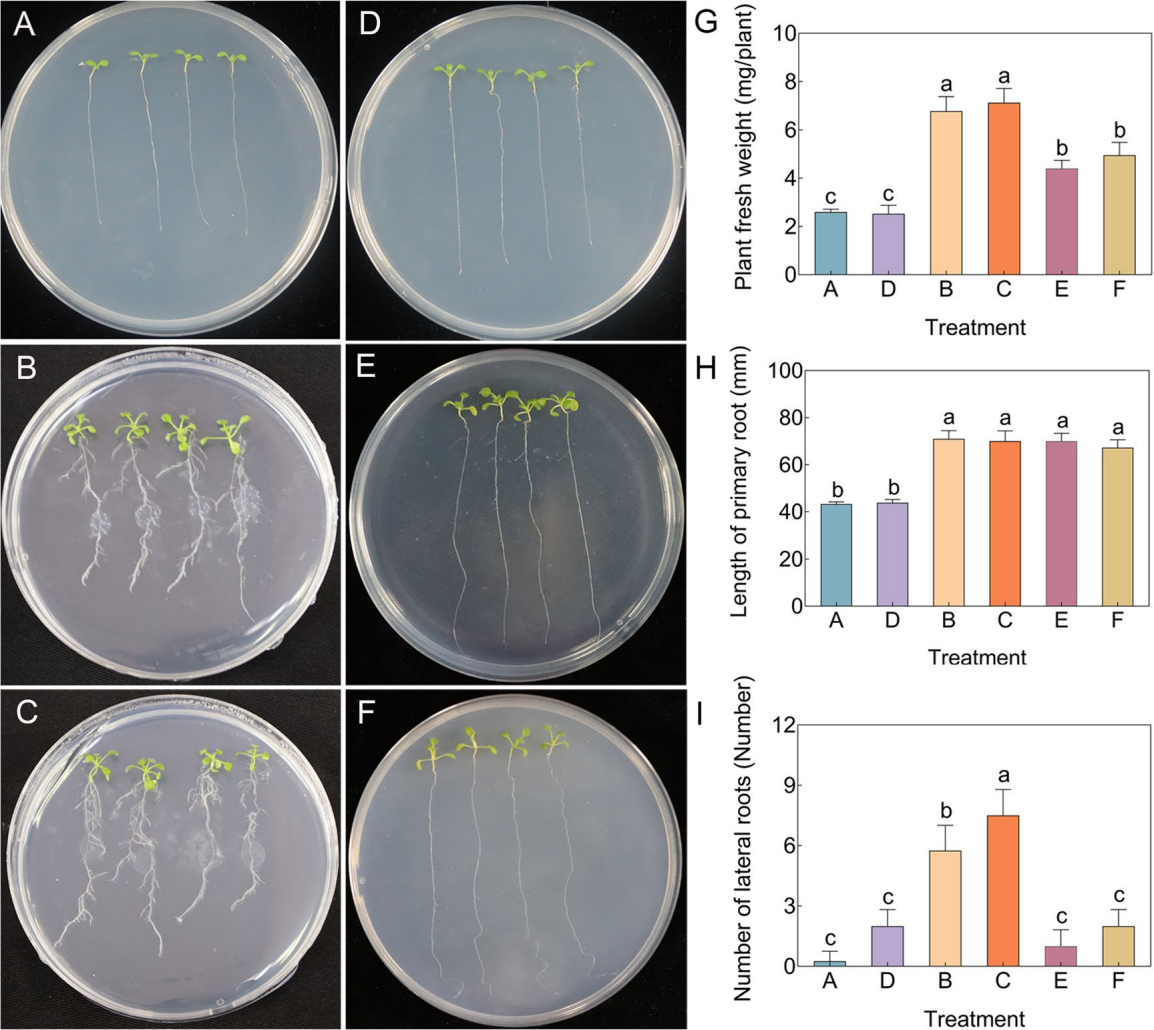
LRB-5 fermentation broth and volatile organic compounds (VOCs) enhance biomass in *Arabidopsis thaliana* Col-0. **A-C** The effect of LRB-5 fermentation broth on the biomass of *Arabidopsis thaliana* Col-0.. A, Sterile distilled water control; B-C: LRB-5 fermentation broth treatment. **D-F** Effect of the VOCs produced by LRB-5 on the biomass of *Arabidopsis thaliana* Col-0. A, Sterile distilled water control; **B**-**C** LRB-5 treatment. **G** Plant fresh weight, **H** Length of primary root, **I** Number of lateral roots. Values in columns followed by the same letter are not significantly different according to Duncan's test at *p* < 0.05. Values are mean ± standard deviation (*n* = 3)

The VOCs and fermentation broth produced by LRB-5 significantly promoted the growth of *Arabidopsis thaliana* (Fig. 2). Compared with the control plants, the length of the primary root exposed to VOCs was 1.57 times higher. The fresh weight of plants treated with fermentation broth increased by 167.31%, and the number of lateral roots increased by 62.5 times. The effects of nine pure compounds identified from LRB-5 were also tested, and the length of the primary root of *Arabidopsis* plants was most significantly enhanced by styrene, heptacosane, 1-tetradecanol, 1-hexadecanol, and heneicosane, with increases of 2.02, 2.67, 1.06, 0.91, and 2.04 times compared to the control, respectively (Fig. S12).

### Disease severity assessment

**Fig. 3:**
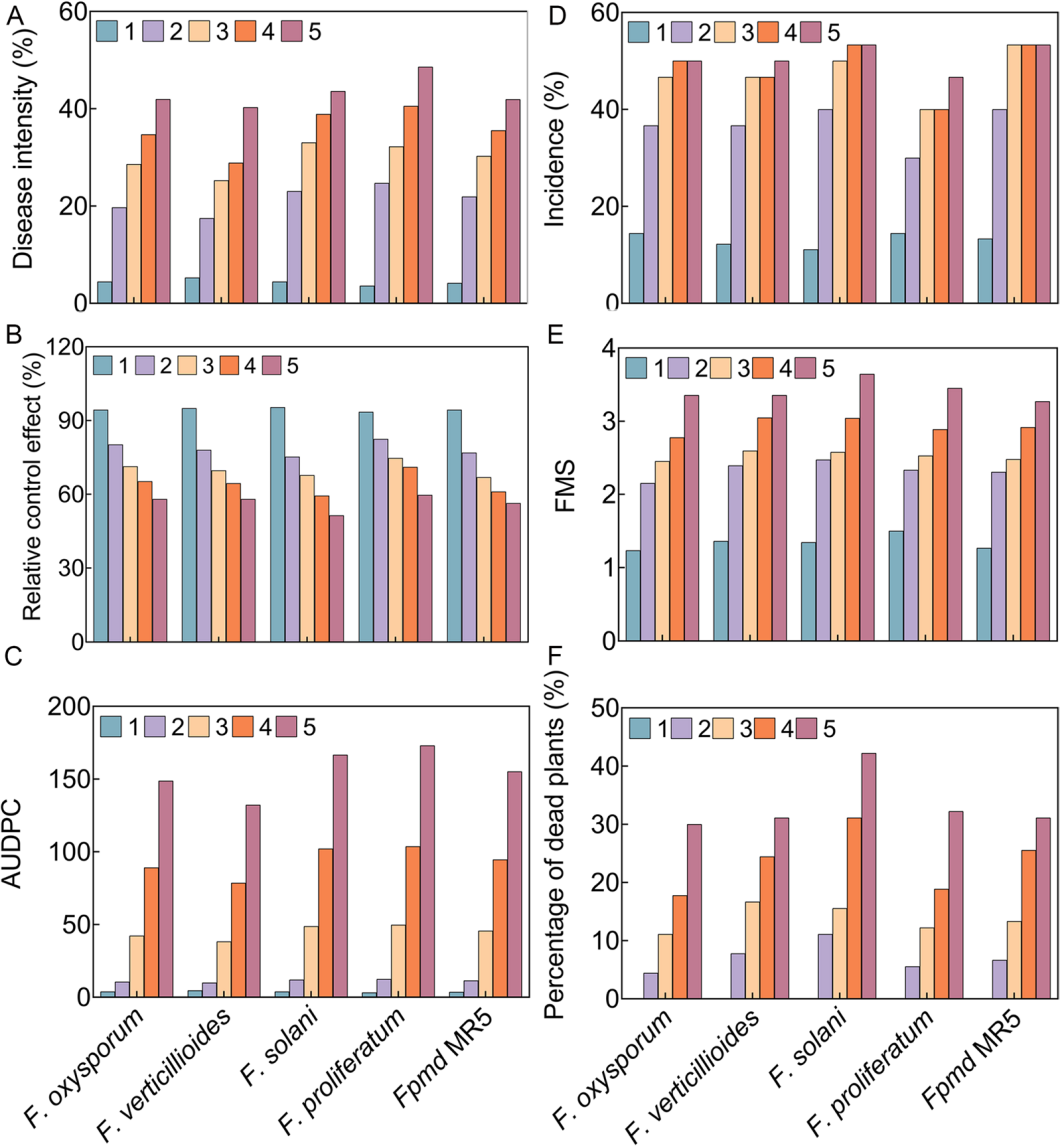
Symptom severity of *Malus hupehensis* Rehd. seedlings from the first to fifth weeks after inoculation with LRB-5. The disease intensity (DI), incidence (percentage of diseased plants), relative control effect, percentage of dead plants (PDP), area under the disease progress curve (AUDPC), and final mean severity of symptoms (FMS) were measured to estimate wilt severity and the ability of plants in different treatment groups to recover from the disease. **A** DI; **B** Relative control effect; **C** AUDPC; **D** incidence; **E** FMS; **F** PDP. Numbers 1, 2, 3, 4, and 5 represent the week. Values are mean ± standard deviation (*n* = 3)

Two weeks after inoculation with *Fusarium*, positive control plants exhibited typical wilt symptoms. Similar symptoms were observed on LRB-5-treated plants but to a significantly lesser extent (Fig. 3, S13). The disease severity index of the positive control plants reached 4.00 in the second week, whereas a reduction in disease progress was noted in plants treated with LRB-5. Analysis of relative AUDPC, incidence, FMS, DI, and PDP showed that LRB-5 significantly reduced wilt symptoms. In the fifth week, the relative control effect of plant seedlings inoculated with LRB-5 was stable at approximately 50–60%. LRB-5 protected plants from *Fusarium* attack throughout the test period. The disease phenotype of the plants was typical of *Fusarium* infection symptoms, such as chlorosis of the leaves, browning from the edge of the leaf followed by rolling and yellowing leaves, wilting and death, black root tips and necrotic rhizodermal, constricted root structure and black necrotic zones, etc. At the end of the experiment, *Fusarium* was successfully re-isolated from the inoculated plants, completing the verification of Koch’s postulates.

### The protective effect of LRB-5 on plant roots

Observation of plant root sections showed that the root epidermal cells infected by *Fusarium* were deformed, broken, detached, and/or irregularly arranged (Fig. S14E, G–I; Fig. S15B–C, E–F). The conidia and hyphae of *Fusarium* appeared in the cortex and vascular column (Fig. S14D–F, I; Fig. S15A, D, C, E). New hyphae were visible mainly close to the cell wall, and mature hyphae were scattered in the cells. There was also a large amount of cell contents (viscous substances and starch granules) in the cortical cells and vascular column, and cauliflower-like structures occurred in the infected root areas (indicated by in Fig. S14G, F; Fig. S15A, D, E–F). The epidermal and cortical cells treated with LRB-5 appeared slightly broken and deformed, but the internal tissue structure remained intact, and the conidia and hyphae of *Fusarium* were attached only to the root epidermal cells (Fig. S14A–C). Root systems from the control treatment were intact, with clear cell boundaries and neatly arranged cells (Fig. S14, Mock).

### Inoculation with LRB-5 promotes the growth and root development of replanted apple seedlings

**Fig. 4:**
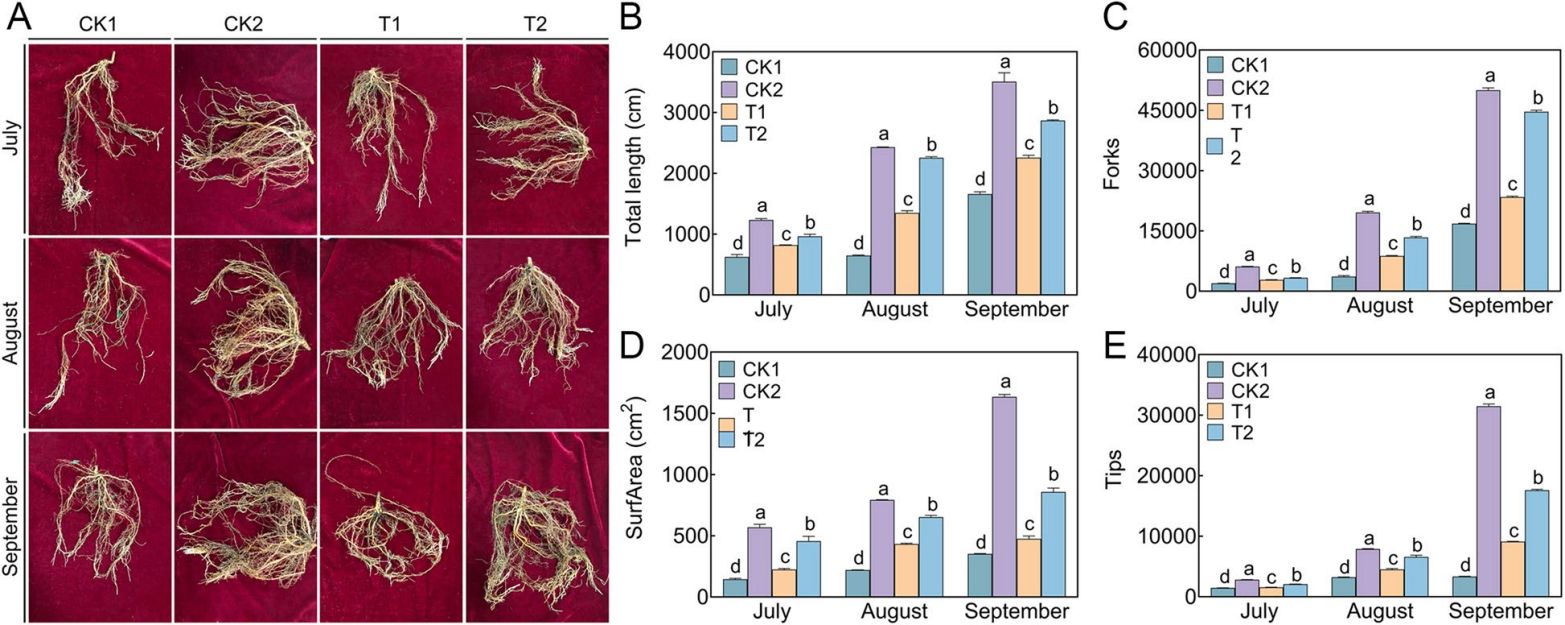
Effect of different treatments on the root architecture of *Malus hupehensis* Rehd. seedlings in July, August and September. **A** Root system scan obtained by an Epson Perfection V850 Pro scanner, each row represents a different month, namely July, August, and September. **B** Total length, **C** Forks, **D** Surface area, **E** Tips. CK1: 31-year-old orchard soil, CK2: Methyl bromide fumigation, T1: Fertilizer carrier, T2: LRB-5 bacterial fertilizer. Values in columns followed by the same letter are not significantly different according to Duncan's test at *p* < 0.05. Values are mean ± standard deviation (*n* = 3)

The application of LRB-5 (T2) significantly promoted the growth of *M. hupehensis* Rehd. seedling roots, and this difference became significant in September. The root length, surface area, number of tips, forks, and vitality increased by 27.11%, 80.48%, 93.22%, 90.60%, and 28.50% in T2 relative to fertilizer carrier (T1), respectively, and by 72.78%, 142.948%, 425.09%, 165.67%, and 48.08% in T2 relative to 31-year-old orchard soil (CK1), respectively (Fig. 4; Table 4). In July, August, and September, the biomass indicators of the T2 treatment were significantly higher than those of the CK1 and T1 treatments. In September, the plant height, ground diameter, fresh weight, and dry weight of T2 were 1.63 times, 1.39 times, 1.54 times, and 2.37 times higher than those of T1, respectively, and 1.87 times, 1.63 times, 2.85 times, and 5.64 times higher than those of CK1, respectively (Table 3).

**Table 3:** Effect of different treatments on seedling biomass of *Malus hupehensis* Rehd. seedlings, including plant height, ground diameter, fresh weight, and dry weight

Treatment	Sampling time	Plant height (cm)	Ground diameter (mm)	Fresh weight (g)	Dry weight (g)
31-year-old orchard soil (CK1)	July	22.27 ± 0.26c	3.79 ± 0.30d	6.47 ± 0.48c	2.08 ± 0.20c
	August	29.79 ± 0.85d	4.08 ± 0.17d	13.42 ± 0.55d	5.19 ± 0.33d
	September	42.25 ± 4.08c	6.44 ± 0.40d	24.23 ± 3.95c	7.27 ± 0.46d
Methyl bromide fumigation (CK2)	July	42.27 ± 2.86a	6.41 ± 0.19a	27.96 ± 2.81a	8.65 ± 1.34a
	August	72.21 ± 2.02a	10.78 ± 0.50a	75.38 ± 5.80a	43.84 ± 1.39a
	September	91.62 ± 7.51a	11.43 ± 0.16a	102.56 ± 20.68a	54.45 ± 7.67a
Fertilizer carrier (T1)	July	25.36 ± 0.91b	4.62 ± 0.15c	13.73 ± 0.79b	4.42 ± 0.56b
	August	38.38 ± 1.12c	6.25 ± 0.13c	27.84 ± 2.15c	8.81 ± 031c
	September	48.58 ± 0.88c	7.54 ± 0.20c	44.72 ± 6.69c	17.27 ± 0.38c
LRB-5 bacterial fertilizer (T2)	July	27.64 ± 0.12b^***^	5.25 ± 0.24b^***^	14.77 ± 0.92b^***^	5.83 ± 0.30b^**^
	August	67.98 ± 1.94b^***^	9.58 ± 0.50b^***^	54.24 ± 2.34b^***^	34.24 ± 0.90b^***^
	September	79.00 ± 0.55b^**^	10.47 ± 0.11b^***^	69.03 ± 1.50b^***^	40.98 ± 1.81b^**^

*ANOVA* Duncan’s multiple range test; different lowercase letters above the columns indicate a significant difference at *p* < 0.05. Values are means ± standard deviation of the mean (*n* = 3). Row comparison (effects of different treatments on plant height, ground diameter, fresh weight and dry weight in July, August and September). Different asterisks indicate significant differences between 31-year-old orchard soil (CK1) and LRB-5 bacterial fertilizer (T2) as defined by Two-sided Student’s t-test

^*^P < 0.05

^**^P < 0.01

^***^P < 0.001

### Effect of LRB-5 on antioxidant enzyme activities

The activities of SOD, POD, and CAT increased from July to September in all treatments (Table 4). In September, the activity of SOD, POD, and CAT increased by 24.80%, 13.85%, and 35.83% in T2 relative to T1, respectively, and by 48.86%, 49.49%, and 41.35% in T2 relative to CK1, respectively. However, the MDA content showed the opposite trend; the MDA content was 23.77%, 30.59%, and 38.01% lower in July, August, and September, respectively, in T2 compared with T1.

**Table 4:** The effects of different treatments on the root vitality and the activities of root protective enzymes (superoxide dismutase (SOD), peroxidase (POD), and catalase (CAT)) and the content of malondialdehyde (MDA) in the root system

Treatment	Sampling time	SOD (U^.^g^−1^)	POD (U^.^g^−1^)	CAT (nmol^.^min^−1.^g^−1^)	MDA (nmol^.^g^−1^)	Respiration rate of root (µmolO_2_·g^−1^FW·min^−1^)
31-year-old orchard soil (CK1)	July	127.87 ± 0.64d	169.73 ± 0.83d	33.12 ± 0.21d	34.98 ± 0.04a^***^	154.68 ± 1.75d
	August	160.21 ± 1.62d	196.00 ± 0.40d	40.16 ± 0.08d	32.65 ± 0.37a^***^	453.31 ± 4.07d
	September	197.70 ± 1.34d	210.40 ± 0.40d	47.92 ± 0.08d	36.36 ± 0.17a^***^	696.13 ± 3.19d
Methyl bromide fumigation (CK2)	July	242.68 ± 1.34a	274.80 ± 1.06a	52.24 ± 0.08a	16.28 ± 0.09d	441.78 ± 2.51a
	August	288.73 ± 1.62a	324.93 ± 0.61a	66.05 ± 0.17a	15.40 ± 0.14d	795.53 ± 4.22a
	September	315.72 ± 0.38a	377.87 ± 0.61a	73.97 ± 0.12a	12.88 ± 0.50d	1176.64 ± 13.00a
Fertilizer carrier (T1)	July	159.36 ± 0.64c	206.93 ± 0.61c	37.31 ± 0.30c	27.06 ± 0.32b	201.31 ± 5.94c
	August	185.06 ± 1.70c	241.07 ± 0.61c	44.45 ± 0.20c	26.34 ± 0.23b	543.12 ± 2.48c
	September	235.82 ± 1.29c	276.27 ± 0.61c	49.87 ± 0.17c	25.64 ± 0.24b	794.33 ± 1.48c
LRB-5 bacterial fertilizer (T2)	July	206.48 ± 0.99b^***^	244.40 ± 0.40b^***^	42.72 ± 0.08b^***^	20.62 ± 0.15c	342.07 ± 1.42b^***^
	August	249.75 ± 1.62b^***^	290.93 ± 0.83b^***^	56.05 ± 0.12b^***^	18.28 ± 0.36c	665.06 ± 1.73b^***^
	September	294.30 ± 1.29b^***^	314.53 ± 1.51b^***^	67.73 ± 0.12b^***^	15.90 ± 0.17c	1021.79 ± 1.98b^***^

*ANOVA* Duncan’s multiple range test; different lowercase letters above the columns indicate a significant difference at *p* < 0.05. Values are means ± standard deviation of the mean (*n* = 3). Row comparison (effects of different treatments on the root vitality and the activities of root protective enzymes in July, August and September). Different asterisks indicate significant differences between 31-year-old orchard soil (CK1) and LRB-5 bacterial fertilizer (T2) as defined by Two-sided Student’s t-test

^*^P < 0.05

^**^P < 0.01

^***^P < 0.001

### Inoculation with LRB-5 improves the microbial environment of replanted soil

**Fig. 5:**
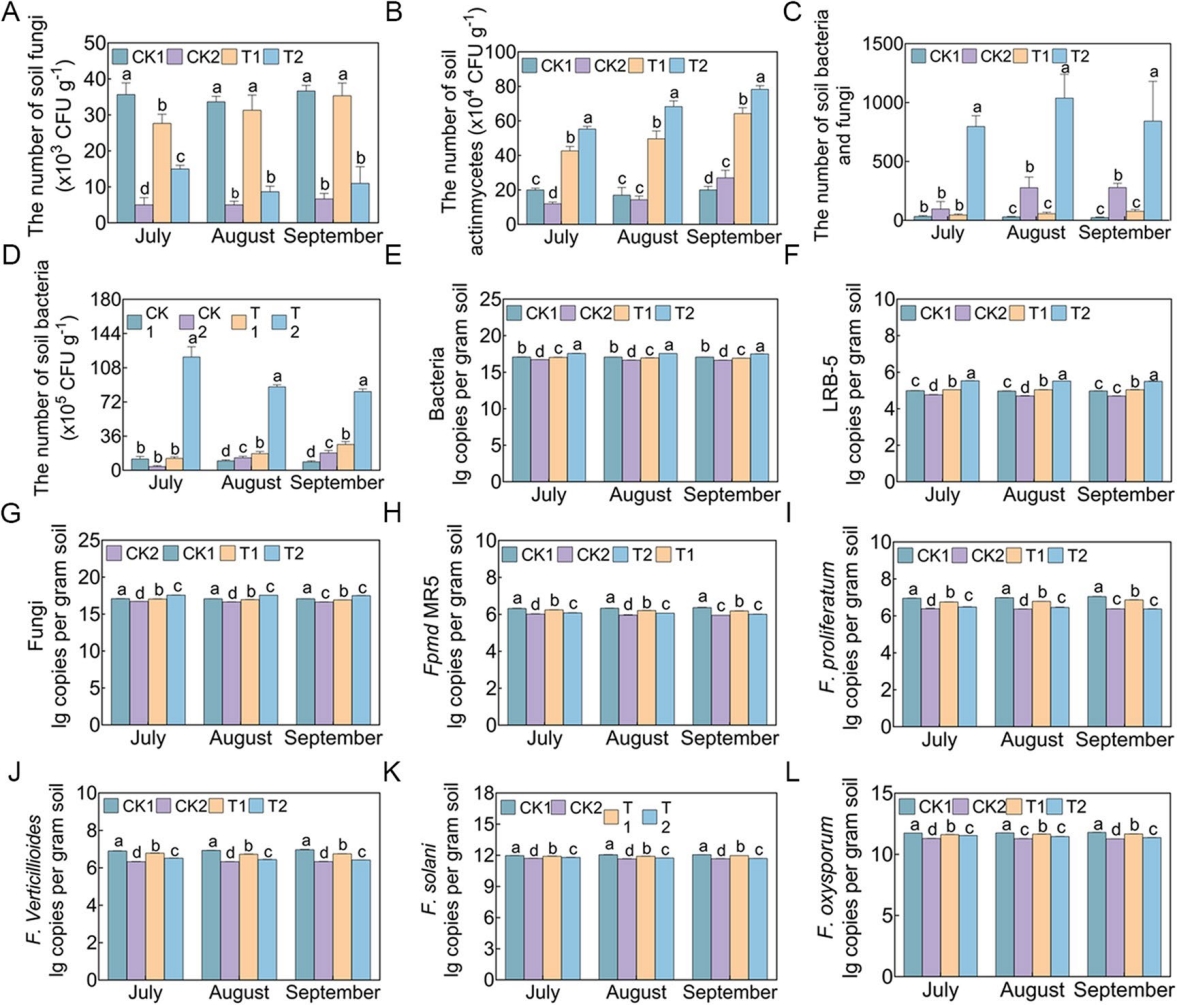
The effects of different treatments on rhizosphere soil microorganisms of apple seedlings in July, August and September. **A-D** The effect of LRB-5 on the density of microorganisms in the rhizosphere of *M. hupehensis* Rehd. seedlings. **A** The number of soil fungi, **B** The number of soil actinmycetes, **C** The number of soil bacteria and fungi, **D** The number of soil bacteria. **E–F** The abundance of (**D**) bacteria, (**G**) fungi, **H**–**L** *Fusarium*, and **F** LRB-5 (log target copy number; *n* = 3) in rhizosphere soils of different treatments. CK1: 31-year-old orchard soil, CK2: Methyl bromide fumigation, T1: Fertilizer carrier, T2: LRB-5 bacterial fertilizer. Values in columns followed by the same letter are not significantly different according to Duncan's test at *p* < 0.05. Values are mean ± standard deviation (*n* = 3)

After T2 treatment, the number of soil bacteria and actinomycetes increased significantly in July, August, and September, and the number of soil fungi decreased significantly (Fig. 5A–D). In September, the number of soil bacteria was 3.04-fold higher in T2 than in T1 and 9.22-fold higher in T2 than in CK1. The number of soil fungi was reduced by 81.82% and 70.00% in methyl bromide fumigation (CK2) and T2 compared with CK1, respectively. The soil bacterial/fungal ratio of T2 in July, August, and September was significantly higher than those of the other treatments. Additionally, qPCR showed that the abundance of *Fusarium* species was significantly reduced in July, August, and September in CK2 and T2 compared with CK1. In September, the abundances of *F. proliferatum*, *F. verticillioides*, *F. oxysporum*, *F. solani*, and *Fpmd* MR5 were 9.56%, 7.83%, 3.58%, 2.98%, and 5.58% lower in T2 than in CK1. Total bacterial and fungal community quantification showed that significantly more bacteria and fewer fungi were detected in July, August, and September in T2 than in CK1 (Fig. 5D, G). Moreover, the abundance of the biocontrol agent *B. vallismortis* LRB-5 remained relatively stable in rhizosphere soil in July, August, and September (Fig. 5F).

Average well color development (AWCD) was used as an indicator of soil microbial activity (Fig. S16). In September, AWCD increased as the incubation time increased for all treatments. T2 had a higher AWCD value than did the other treatments, indicating that the addition of LRB-5 increased the activity of microorganisms. The utilization rates of six substrate groups (i.e., polymers, carbohydrates, miscellaneous, amines/amides, carboxylic acids, and amino acids) by LRB-5 (T2) were significantly higher than in the other treatments, with increases of 45.65%, 13.11%, 42.50%, 17.42%, 14.16%, and 25.93% compared to T1, respectively. The utilization efficiency of CK2 for the six carbon sources was significantly lower than those of the other treatments.

**Fig. 6:**
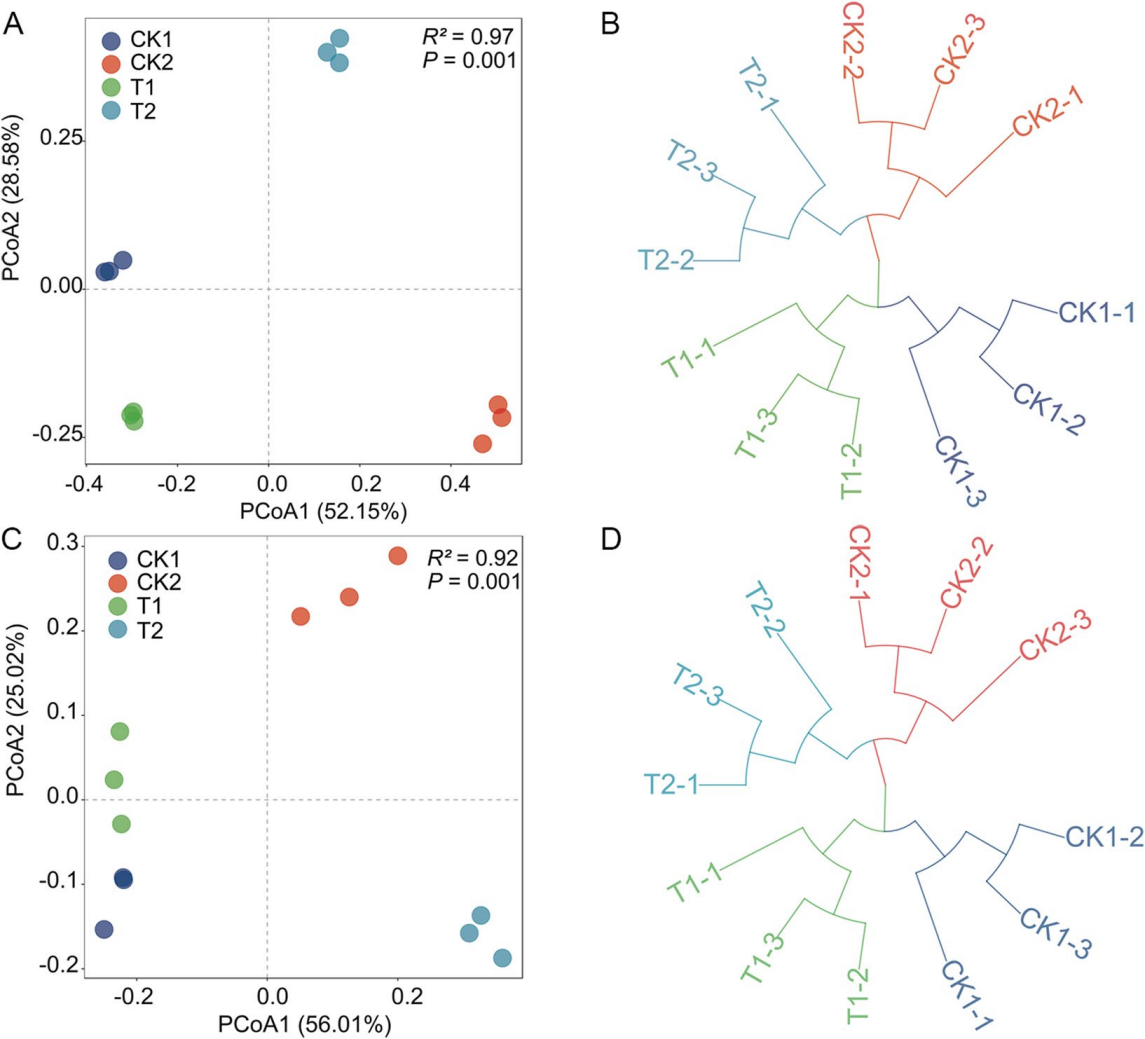
Microbial community structures in the different treatments based on the T-RFLP data. Principal coordinate analysis (PCoA) (**A**, **C**) and cluster analysis (**B**, **D**) plot based on the OTUs of Bray–Curtis distance. The *P* values were from PERMANOVA. **A**-**B** (bacteria) and **C**-**D** (fungi). CK1: 31-year-old orchard soil, CK2: Methyl bromide fumigation, T1: Fertilizer carrier, T2: LRB-5 bacterial fertilizer

Principal component and cluster analyses showed that there were significant differences in soil fungal community structure between T2 and CK1, and the soil bacterial community structure of T2 was significantly different from that of the other treatments (Fig. 6). The Margalef’s, Shannon, and Brillouin indices of soil fungal communities increased significantly in T2 relative to CK1, by 1.47%, 4.46%, and 53.78%, respectively. Meanwhile, the Simpson’s and McIntosh indices were significantly lower in T2 than in both CK1 and T1 (Table S10). The changes in soil bacterial communities showed the opposite trend. The Simpson’s and McIntosh indices of soil bacterial communities increased in T2 relative to CK1, by 18.85% and 47.74%, respectively. Conversely, the Margalef’s, Shannon, and Brillouin indices were lower in T2 than in CK1.

### Inoculation with LRB-5 increases soil enzyme activity

The activities of catalase, acid phosphatase, sucrase, and urease significantly increased from July to September in T2, T1, and CK2 conditions. Relative to T1, the urease activity of T2 in July, August, and September was 1.37-fold, 1.53-fold, and 1.57-fold higher, respectively; the acid phosphatase activity was 1.03-fold, 1.05-fold, and 1.19-fold higher, respectively; the sucrase activity was 1.06-fold, 1.17-fold, and 1.33-fold higher, respectively; and the catalase activity was 1.10-fold, 1.17-fold, and 1.19-fold higher, respectively. In September, the activities of catalase, acid phosphatase, sucrase, and urease increased by 32.89%, 33.84%, 62.06%, and 168.95%, respectively, in T2 relative to CK1 (Table 5).

**Table 5:** The effects of different treatments on soil enzyme activities, including catalase (S-CAT), acid phosphatase (S-ACP), sucrase (S-SC), and urease (S-UE)

Treatment	Sampling time	S-CAT (μmol^.^d^−1.^g^−1^)	S-UE (μg^.^d^−1.^g^−1^)	S-SC (mg^.^d^−1.^g^−1^)	S-ACP (μmol^.^d^−1.^g^−1^)
31-year-old orchard soil (CK1)	July	21.12 ± 0.03b	82.62 ± 2.20c	21.24 ± 0.06c	3.68 ± 0.01c
	August	19.52 ± 0.07d	76.21 ± 0.87d	20.57 ± 0.23d	3.65 ± 0.01d
	September	19.56 ± 0.24d	72.43 ± 2.52d	20.17 ± 0.20d	3.64 ± 0.01d
Methyl bromide fumigation (CK2)	July	19.50 ± 0.32d	72.43 ± 2.67d	20.46 ± 0.05d	3.65 ± 0.02c
	August	20.52 ± 0.06c	81.46 ± 2.31c	21.02 ± 0.10c	3.69 ± 0.017c
	September	21.34 ± 0.11c	86.41 ± 2.52c	21.60 ± 0.07c	3.73 ± 0.03c
Fertilizer carrier (T1)	July	20.64 ± 0.21c	92.53 ± 2.81b	21.87 ± 0.07b	3.77 ± 0.02b
	August	21.03 ± 0.04b	116.42 ± 2.32b	22.70 ± 0.03b	3.97 ± 0.03b
	September	21.88 ± 0.02b	124.28 ± 0.00b	24.54 ± 0.33b	4.10 ± 0.07b
LRB-5 bacterial fertilizer (T2)	July	22.67 ± 0.04a^***^	126.32 ± 3.07a^***^	23.15 ± 0.22a^**^	3.90 ± 0.02a^***^
	August	24.61 ± 0.17a^***^	178.18 ± 1.82a^***^	26.59 ± 0.25a^***^	4.15 ± 0.02a^***^
	September	25.99 ± 0.02a^***^	194.79 ± 1.82a^***^	32.69 ± 0.72a^***^	4.87 ± 0.03a^***^

*ANOVA* Duncan’s multiple range test; different lowercase letters above the columns indicate a significant difference at *p* < 0.05. Values are means ± standard deviation of the mean (*n* = 3). Row comparison (effects of different treatments on soil enzyme activities in July, August and September). Different asterisks indicate significant differences between 31-year-old orchard soil (CK1) and LRB-5 bacterial fertilizer (T2) as defined by Two-sided Student’s t-test

^*^P < 0.05

^**^P < 0.01

^***^P < 0.001

### Inoculation with LRB-5 reduces phenolic acid content in replanted soil

In July, August, and September, the soil phenolic acid content (including cinnamic acid, phlorizin, benzoic acid, ferulic acid, *p*-hydroxybenzoic acid, syringic acid) was highest in CK1 and T1 conditions. In September, the soil contents of cinnamic acid, phlorizin, benzoic acid, ferulic acid, *p*-hydroxybenzoic acid, and syringic acid were 55.65%, 58.41%, 62.58%, 75.69%, 71.10%, and 63.62% lower, respectively, in T2 than in CK1 (Fig. S17).

## Discussion

In recent years, biological control agents (BCAs) have gradually become valuable alternatives to chemical fungicides owing to their broad-spectrum antimicrobial properties, safety, and environmental friendliness, and they are widely used in agriculture to control fungal diseases (Duan et al. 2022a). Bacteria, such as *Streptomyces*, *Pseudomonas*, and *Bacillus*, are key producers of antimicrobial substances, with *Bacillus* spp. receiving considerable attention for their potential in biological control owing to their rapid growth and ability to synthesize many secondary metabolites (Chen et al. 2020; Etesami et al. 2023; Granada and Skariyachan 2025; Li et al. 2024, 2025). Antagonistic *Bacillus* spp., such as *Bacillus amyloliquefaciens*, *Bacillus vallismortis*, *Bacillus velezensis*, and *Bacillus subtilis*, have been isolated from various environments (Chen et al. 2022; Duan et al. 2022a; Marković et al. 2023; Wu et al. 2019). However, *Bacillus vallismortis* has been widely used to control various diseases in crops, such as potatoes, tomatoes, rices, and peppers, and has shown some efficacy in controlling diseases in fruit crops, such as apples and pomegranates (Thanh et al. 2009; Thepbandit et al. 2023; Teli 2024; Xu et al. 2023). In the context of ARD, only one related study has been published, and it was reported that *B. vallismortis* HSB-2 can antagonize the main pathogenic fungi of ARD, significantly promote the growth of *M. hupehensis* Rehd. seedlings, enhance soil enzyme activity, and thus alleviate ARD (Duan et al. 2022a). In the present study, we isolated *B. vallismortis* LRB-5 from the roots of healthy apple trees in a replanted orchard severely affected by ARD and validated its ability to control ARD through pot experiments. Thus, inoculation with LRB-5 (T2) significantly increased the biomass of *M. hupehensis* Rehd. seedlings and reduced the incidence of ARD caused by *Fusarium* species. This may be explained by the ability of LRB-5 to secrete various growth-promoting substances during root colonization, thus stimulating root growth and thereby promoting the growth and development of the aboveground parts of plants. This relationship will be further investigated in future studies.

Long-term continuous cropping often leads to soil nutrient imbalances (Hou et al. 2021). In the present study, after inoculating apple roots with LRB-5, the total surface area, volume, number of forks, and root tips of the plant roots significantly increased, expanding the range of nutrient and water absorption by the roots. This enhanced the plant’s ability to absorb nutrients and improved its tolerance to ARD (Zhang et al. 2021; Zhang et al. 2022). Additionally, during colonization, LRB-5 can induce the plant roots to produce defense enzymes more rapidly to resist pathogen invasion and activate the plant’s resistance system (Wei et al. 2023). Spore germination is a necessary condition for pathogenic fungi to infect host tissues, so inhibiting spore germination can effectively reduce disease severity (Li et al. 2020; Zhu et al. 2016). PAS staining revealed reduced *Fusarium* infection in roots treated with LRB-5, further demonstrating that it can significantly inhibit *Fusarium* spore germination and form a protective layer in the rhizosphere of apple seedlings, thereby protecting the roots from pathogenic *Fusarium* species associated with ARD. At the same time, the LRB-5 strain secretes cell wall-degrading enzymes, such as proteases and cellulases, and produces siderophores. These substances have been reported to significantly disrupt the structural integrity of fungal cell walls, inhibit pathogen growth, and protect plant roots from infection (Chen et al. 2020; Etesami et al. 2023; Li et al. 2024). Cellulases produced by microorganisms can also act as elicitors of plant immunity (Ma et al. 2015). Siderophores are high-affinity iron-chelating compounds secreted by bacteria, which inhibit pathogen growth by limiting iron availability (Pradhan et al. 2023). Similarly, *B. subtilis* has emerged as a promising biocontrol agent due to its ability to produce siderophores, chitinases, and cellulases (Villa-Rodriguez et al. 2019). In related studies, siderophore-producing *B. velezensis* was isolated and demonstrated to protect tobacco against bacterial wilt caused by *Ralstonia solanacearum* (Etesami et al. 2023).

In the present study, the LRB-5 strain exhibited multiple plant growth-promoting traits, including the synthesis of siderophores, amino acids, IAA, and ammonia, as well as nitrogen fixation and phosphate and potassium solubilization capabilities. Phosphate- and potassium-solubilizing microorganisms help plants better absorb phosphorus (P) and potassium (K) by secreting organic acids and siderophores, and siderophores can chelate metal ions and release phosphates for plant use (Jain et al. 2022; Kashyap et al. 2021; Liu et al. 2019). It has been reported that some amino acids (i.e., leucine, lysine, and glutamic acid) can effectively drive soil microbial activity, induce plant resistance mechanisms, and combat soil-borne diseases (Wen et al. 2021). Nitrogen fixation, ACC deaminase, IAA, and ammonia production can induce plant resistance and promote plant growth (Duan et al. 2022b; Jiang et al. 2023; Penrose and Glick 2003). In this study, LRB-5 strain significantly enhanced the root activity of *M. hupehensis* Rehd. seedlings. These characteristics indicate that the LRB-5 strain not only inhibits pathogens but also promotes overall plant growth through multiple mechanisms.

Soil enzymes are an essential component of soil ecosystems, playing a crucial role in the transformation and cycling of nutrients, such as nitrogen and phosphorus, promoting aggregate formation, enhancing soil stability, and degrading pollutants, like pesticides (Rahul et al. 2022). Studies have shown that *Bacillus* strains can significantly enhance soil enzyme activity, promote the cycling of nitrogen, phosphorus, and potassium, and facilitate the decomposition of organic matter, thereby improving soil quality, enhancing plant resistance to soil-borne pathogenic fungi, and reducing disease incidence (Cui et al. 2019; Duan et al. 2022a, b; Rawat et al. 2016; Wang et al. 2013). Soil enzymes also provide carbon and nitrogen sources for microorganisms by decomposing organic matter, thus improving their environment and supporting their growth and reproduction (Banerjee and Van Der Heijden 2023; Liu et al. 2024). The results of this study indicate that inoculation with LRB-5 (T2) significantly increases the activity of soil phosphatase, sucrase, catalase, and urease, thereby enhancing plant nutrient uptake and disease resistance. These findings were consistent with previous research, confirming that microorganisms can regulate soil enzyme activity to improve nutrient transformation between plant roots and the soil environment, promoting plant growth and enhancing disease resistance (Etesami et al. 2023; Huang et al. 2024; Zhu et al. 2024). Previous studies have found that soil enzyme activity is closely related to the abundance and activity of specific microbial communities. For example, urease and phosphatase activity may be associated with the abundance of Burkholderiaceae and nitrogen-fixing bacteria, while catalase activity is positively correlated with aerobic microbial biomass (Debnath et al. 2015; Fei et al. 2020; Lemanowicz 2011; Li et al. 2012). Therefore, the increase in soil enzyme activity may reflect changes in microbial community composition (Iovieno et al. 2009). At the same time, microbial activity also influences soil enzyme activity. Active microorganisms synthesize and secrete various enzymes, participating in soil biochemical reactions. After microbial death, intracellular enzymes are released into the soil, where they continue to influence soil material transformation and ecological processes (Larsbrink and McKee 2020; Plante and Parton 2007; Zhu 2011). Thus, in healthy soils with abundant microorganisms, soil enzyme activity is typically relatively high (KaliPrasanna and Narasimha 2012; Mganga et al. 2015). In summary, LRB-5 may promote plant growth by increasing soil enzyme activity, accelerating the decomposition and cycling of soil nutrients, and optimizing the structure of soil microbial communities.

Studies have shown that phenolic acids in root exudates or decomposition residues are one of the important causes of ARD (Bai et al. 2019; Wang et al. 2014). Replanted soils often have high concentrations of phenolic acids (such as benzoic acid, cinnamic acid, and *p*-hydroxybenzoic acid), which can damage the antioxidant system of apple seedling roots, reduce photosynthesis and transpiration rates, and inhibit seedling growth (Bai et al. 2019; Gao et al. 2010; Wang et al. 2014; Yin et al. 2016). Additionally, phenolic acids can promote the growth and spore formation of pathogenic *Fusarium* in the soil, further inhibiting plant growth (Yin et al. 2017; Zhang et al. 2021). This study found that the LRB-5 strain significantly reduced the content of cinnamic acid, phlorizin, benzoic acid, ferulic acid, *p*-hydroxybenzoic acid, and syringic acid in the soil and decreased the relative abundance of *Fusarium*. Similar phenomena have been reported in other studies (Duan et al. 2022a,b; Ling et al. 2010). The degradation of phenolic acids by rhizosphere microorganisms is an effective means of alleviating ARD (Zhang et al. 2010). Therefore, the reduction in phenolic acid content may be closely related to the enrichment of phenolic acid-degrading microorganisms (such as *Pseudomonas*, *Staphylococcus*, *Streptomyces*, *Bacillus subtilis*, and *Trichoderma*) in the rhizosphere, thereby improving the structure of the soil microbial community (Bai et al. 2019; Chen et al. 2011; Nogales et al. 2011; Zhou et al. 2012, 2018). This suggests that LRB-5 may promote plant growth by enriching beneficial microbial communities that degrade phenolic acids, directly reducing damage to plant roots, and/or indirectly inhibiting the growth of *Fusarium* spp.

Microbial activity plays a crucial role in the cycling of soil nutrients, such as nitrogen, phosphorus, and carbon, the accumulation and transformation of organic matter, promoting plant growth, suppressing pathogens, and maintaining ecological balance (Bhatti et al. 2017; Xiang et al. 2021). Numerous studies have shown that long-term continuous cropping shifts the soil microbial community structure from being bacteria-dominated to fungi-dominated (He et al. 2023; She et al. 2017). This shift disrupts the balance of the soil microbial community, enabling an increase in harmful pathogens and a decline in beneficial microbial populations, thereby reducing soil health (Liu et al. 2024; Wang et al. 2018). In the present study, inoculation with LRB-5 significantly promoted bacterial diversity and carbon source utilization, reduced fungal dominance and abundance, and increased the ratio of bacteria to fungi in the soil. Additionally, the abundance of LRB-5 in the soil remained at a high level, enabling it to stably colonize the root zone of plants. By producing volatile antimicrobial substances, LRB-5 effectively inhibited the growth and reproduction of pathogenic *Fusarium*, thus alleviating ARD. The increase in beneficial microorganisms and the reduction in pathogenic fungi in the soil gradually transformed the replanted soil into a healthier state (Li et al. 2025; Wang et al. 2013, 2024). Qiao et al. (2024) developed a synthetic microbial community that can suppress soil-borne pathogenic fungi, recruit antagonistic bacteria and growth-promoting microorganisms, improve the soil environment, and thereby promote plant growth. Recent research has shown that *B. velezensis* can increase the abundance of beneficial microorganisms (e.g., *Pseudomonas*, *Bacillus*, *Arthrobacter*, *Mortierella*, *Claroideoglomus*, and *Rhizophagus*) in the rhizosphere soil by secreting metabolites after forming biofilms on plant roots, thereby enhancing the plant’s resistance to *Fusarium* invasion (Sun et al. 2022; Wei et al. 2023). Therefore, we speculate that LRB-5 may recruit some beneficial microorganisms with synergistic effects, and this process will be a focus of our future research. Devi et al. (2022) found that the combined application of *Bacillus* (*B. velezensis* and *Bacillus* sp*.*) and arbuscular mycorrhizal fungi (*Funneliformis mosseae* and *Glomus fasciculatum*) significantly improved the control of tomato wilt, demonstrating a clear synergistic effect, further reducing the disease index and mitigating damage to tomato plants. Santos et al. (2022) demonstrated that *Bacillus velezensis* can be effectively combined with organic mineral fertilizers to reduce phosphate dosage and increase sugarcane biomass. Thus, LRB-5 could be integrated with other control measures as a promising strategy for sustainable apple cultivation, reducing reliance on chemical fertilizers, and promoting soil health.

Our study also found that the LRB-5 strain exhibits broad-spectrum antagonistic activity against various plant pathogens. Many previously identified *Bacillus* strains have similarly exhibited excellent antagonistic activity against pathogens through producing antimicrobial substances (Chen et al. 2020; Etesami et al. 2023; Li et al. 2024). For example, *B. vallismortis*, which produce numerous antimicrobial bioactive metabolites, such as thermostable alkalophilic cellulose, bacillomycin D, surfactins, iturins, and fengycins, has been tested for its biocontrol properties (Thepbandit et al. 2023; Xu et al. 2023). The cell-free culture filtrate of LRB-5 also exhibited antifungal activity, indicating its ability to produce secondary metabolites such as antimicrobial peptides, thus significantly inhibiting abnormal hyphal morphology (e.g., elongation, increased branching, deformation) and spore germination of *Fusarium* pathogens associated with ARD. Similar phenomena have been observed in other studies (Duan et al. 2022a, b; Fazle Rabbee and Baek 2020). *Bacillus* species typically contain gene clusters encoding proteins involved in the synthesis of antifungal lipopeptides and polyketides, such as surfactin and bacillomycin, which are key features enabling their as biocontrol agents, playing a role in suppressing the growth of pathogenic fungi in the plant rhizosphere (Chowdhury et al. 2015; Duan et al. 2022b; Li et al. 2024a). Granada and Skariyachan (2025) found that antifungal substances (i.e., β-amyrin and dihydroxy octadecenoic acid) secreted by *Bacillus velezensis* CBMB205 interact with target proteins (chitin synthase 1 and β−1,3-glucan synthase) of *F. oxysporum*, serving as the molecular mechanism of its antifungal activity. Thus, LRB-5 may also produce the aforementioned antifungal compounds, not only effectively resisting *Fusarium*-induced ARD but also demonstrating substantial potential for controlling a range of other plant fungal diseases.

Rhizosphere microorganisms are ideal biocontrol agents for soil-borne pathogens, as the rhizosphere is the first line of defense for plants against pathogens and is also crucial for building healthy root structures and supporting plant growth (Li et al. 2024). After treatment with the LRB-5 strain, disease severity was significantly reduced, and plant height and other growth indicators improved markedly. This suggests that the LRB-5 strain may enhance plant resistance to soil-borne pathogens and promote growth by improving the rhizosphere microbial community, a phenomenon also reported in other studies (Duan et al. 2021; Geng et al. 2022). Apple trees are perennial plants, and long-term cultivation leads to the accumulation of pathogenic fungi (e.g., *Fusarium*) associated with ARD in the rhizosphere, complicating control of ARD (Duan et al. 2022c; Li et al. 2025). Ideal biocontrol agents (BCAs) possess the ability to colonize soil or roots over the long term to suppress or control pathogen populations (Duan et al. 2021, 2022b; Liu et al. 2019; Wu et al. 2019). The present study found that the LRB-5 strain indeed stably colonized the rhizosphere of *M. hupehensis* Rehd. seedlings, significantly reducing the disease index of *Fusarium*-induced ARD, with a control efficacy exceeding 50%. This indicates that the growth-promoting and pathogen-suppressing abilities of the LRB-5 strain are closely related to its colonization capacity. Stable and persistent colonization of the apple rhizosphere is integral to the effective biocontrol performance of LRB-5.

In recent years, many studies have found that VOCs produced by certain biocontrol agents can not only inhibit pathogens but also promote plant growth and induce systemic resistance in plants (Duan et al. 2022a; Wu et al. 2019). In the present study, LRB-5 was confirmed to produce 25 volatile compounds, including alcohols, ketones, organic acid esters, and alkane derivatives. These are similar to the VOCs previously reported to be produced by *Bacillus* spp. (Duan et al. 2022a; Tang et al. 2024; Wu et al. 2019). Among the identified VOCs, nine pure compounds (5-methyl-2-heptanone, heptacosane, heneicosane, 1-tetradecanol, 1-hexadecanol, 2,5-dihydroxybenzaldehyde, 2-ethyl-1-hexanol, styrene, and toluene) exhibited varying levels of antimicrobial activity against all tested plant pathogens, with inhibition rates of approximately 70%. In a recent study by Duan et al. (2022a), *n*-pentadecanol, eicosane, 2-undecanone, dodecane, butylated hydroxytoluene, and 6-methyl-2-heptanol produced by *B. vallismortis* HSB-2 significantly inhibited pathogenic fungi. Similarly, Tang et al. (2024) found that the VOCs 2-dodecanone and 2-undecanone produced by *Bacillus velezensis* LT1 inhibited the pathogen *Sclerotium rolfsii* by 81.67% and 80.08%, respectively. Dutta et al. (2025) demonstrated that VOCs (e.g., acetoin, carbon dioxide, long-chain decanes, benzaldehyde, and dimethyl disulfide) emitted by *B. vallismortis* EXTN-1 in the promotion of tobacco growth through the short-term priming of seeds or seedlings. In the present study, VOCs, such as styrene, heptacosane, 1-tetradecanol, 1-hexadecanol, and hexacosane, produced by LRB-5 were found to promote the growth of both shoots and root systems of *Arabidopsis* plants. Similar findings have been reported in other studies, including the observations of acetoin and 2,3-butanediol produced by *B. amyloliquefaciens* L3, albuterol and 1,3-propanediol produced by *B. subtilis* SYST2, 2-heptanol, 6-methyl-2-heptanol, tetratetracontane, tetradecane, and dodecane produced by *B. vallismortis* HSB-2, and 2-pentylfuran produced by *B. megaterium* XTBG34 all exhibiting plant growth-promoting activity (Duan et al. 2022a; Ryu et al. 2003; Tahir et al. 2017; Wu et al. 2019; Zou et al. 2010). In the present study, LRB-5 treatment significantly enhanced plant vigor and promoted lateral root development, likely due to its volatile organic compounds suppressing pathogens and inducing plant defense responses. This aligns with the findings of Wang et al. (2021), who demonstrated that microbial volatile organic compounds can positively influence plant growth by modulating plant-pathogen interactions and activating defense mechanisms.

Biofertilizers represent a promising tool for sustainable agricultural intensification (Kumar et al. 2022), yet their commercialization faces challenges due to inconsistent field efficacy and stability, with successful adoption depending on advanced formulation and application methods (Fadiji et al. 2024; Nakachew et al. 2024). In this study, the LRB-5 bacterial fertilizer was produced via solid-state fermentation (cow dung: straw = 3:1), which creates a protective nutrient-rich environment for microbial inoculants, enhances storage efficiency, and offers advantages including low-cost substrates, sustainable production, minimal waste generation, and improved microbial survival (Duncker et al. 2021; Duan et al. 2021). However, inoculants combined with solid carriers still face limited shelf life, necessitating innovative stabilization approaches (Santhosh 2015). Optimal fertilizer placement methods (e.g., band placement and localized placement in planting holes) can reduce nutrient loss and improve uptake (Nkebiwe et al. 2016). Raimi et al. (2017) demonstrated that biofertilizers offer higher cost-effectiveness compared to other nutrient management strategies, particularly inorganic fertilizers. For field applications, LRB-5 (400–460 USD/acre) proves more economical than common fumigants such as dimethyl disulfide (DMDS; 60 g/m^2^), methyl bromide (568.50 USD/acre), and 1,3-dichloropropene (600 USD/acre), thereby improving farmers'profitability (Duan et al. 2021). In September, LRB-5 and methyl bromide fumigation showed nearly identical efficacy in promoting seedling growth, indicating LRB-5 has the potential to become an excellent biological control agent to replace fumigants to control ARD.

## Conclusions

**Fig. 7:**
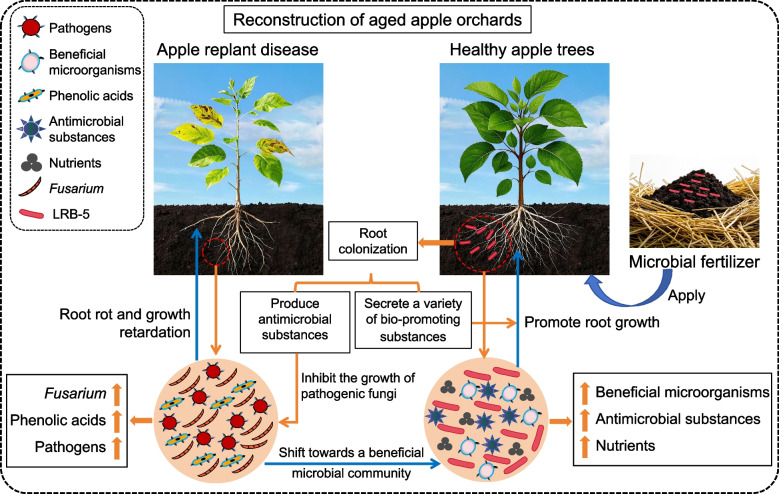
Conceptual model displaying the potential role of LRB-5 in influencing the pathogen density (qPCR) in the soil and the occurrence of ARD

We isolated a high-quality biocontrol strain, *Bacillus vallismortis* LRB-5, from the roots of a healthy apple tree in an orchard containing trees with ARD. This strain effectively alleviated ARD. The metabolites of LRB-5 significantly inhibited the mycelial growth and spore germination of *Fusarium*, and the VOCs it produced not only suppressed various plant pathogens but also promoted plant root growth. Additionally, LRB-5 was able to stably colonize the rhizosphere soil of plants, significantly reducing the abundance of *Fusarium*, the carbon source utilization level of fungi, and the dominance index, while also decreasing the content of phenolic acids in the soil. LRB-5 further enhanced soil enzyme activity, increased the soil bacteria/fungi ratio, and improved the soil microbial environment (Fig. 7). Future research is required to further validate the efficacy of the LRB-5 strain in controlling ARD under field conditions, to optimize its fermentation conditions to enhance its inhibitory effect against plant pathogens, and to accelerate its commercialization in agricultural production.

## Materials and methods

### Sample collection

In May 2016, samples were collected from orchards in Lutou Town, Longkou City, Shandong Province (longitude: 120.465274; latitude: 37.606707), which had been replanted for 5 years on old apple orchards (over 25 years old). The orchards used *Malus* × *robusta* (Carrière) Rehder as the rootstock and *Malus pumila* Mill as the scion. In small localized plots, plants exhibited typical symptoms of replant disease (reduced plant vitality, yellowing of leaves, twig growth retardation, or death), while the rest of the orchard consisted of trees showing optimal vigor (Fig. S1). Soil was collected within the row (10 to 20 cm deep in a 30-cm-diameter area with the trunk at the center, from five sites per orchard) from the root zone of trees displaying optimal growth and high-quality production standards (asymptomatic trees). Their roots, stems, leaves, and fruits were collected using sterile spatulas. After sieving, mixing, and removing impurities, the soil was sealed in bags, and the basic information of the collection site was recorded. The samples were packed in clean, dry, sterile polyethylene bags, placed on dry ice in the field, and transported as quickly as possible to the National Key Laboratory of Crop Biology for bacterial isolation. Samples for DNA extraction were stored at −80°C, samples for bacterial isolation were stored at −4°C, and the remaining soil was sieved (50-mesh) for the determination of soil physicochemical properties (Duan et al. 2022c). The remaining bulk orchard samples were stored moist in totes lined with plastic at 5°C and used for ARD bioassays in the greenhouse (Xiang et al. 2021).

### Plant materials

In March 2017, the seeds of *Malus hupehensis* Rehd. were stratified at 4°C for approximately 30 days. Seeds with full buds and cracks were selected and sown in 50-hole seedling trays filled with seedling substrate (organic matter ≥ 45%, pH 5.8–6.5, EC 1.0–1.5 mS·cm⁻^1^). After one month, seedlings with uniform growth were transplanted into 32 cm × 25 cm flower pots. After 30 days of cultivation, disease-free plants with consistent growth were selected for further planting.

### Plant pathogens, growth conditions, and inoculum preparation

The *Alternaria alternata*, *Albifimbria verrucaria*, *Aspergillus flavus*, *Phytophthora cactorum*, *Phoma macrostoma*, *Penicillium brasilianum*, *Rhizoctonia solani*, *Valsa mali*, *F. oxysporum*, *F. verticillioides*, *F. proliferatum*, *Fpmd* MR5, and *F. solani* used in this study were preserved and provided by the National Key Laboratory of Crop Biology, College of Horticultural Science and Engineering, Shandong Agricultural University. Among them, *A. alternata*, *A. verrucaria*, *A. flavus*, *P. macrostoma*, *P. brasilianum*, *F. oxysporum*, *F. verticillioides*, *F. proliferatum*, *Fpmd* MR5, and *F. solani*, isolated from apple replant soil, were highly pathogenic to *Malus hupehensis* Rehd. seedlings (Duan et al. 2022c; Wang et al. 2018). The pathogen cultures were grown on potato dextrose agar (PDA; 200 g potato, 5 g beef extract, 20 g glucose, 20 g agar, pH 7.0) for 7 days at 28°C. The *Fusarium* spore suspension (10⁶ conidia·mL⁻^1^) was prepared according to the method described by Duan et al. (2021).

### Antagonistic bacteria

#### Isolation of antagonistic bacteria

Antagonistic bacteria were isolated from the collected samples using the methods of Duan et al. (2021). Tissue grinding fluid and soil samples were serially diluted with sterile water, spread (100 μL) onto Trypticase Soy Agar (TSA: casein tryptone 15 g, soy peptone 5 g, NaCl 5 g, agar 15 g, pH 7.3 ± 0.2), and incubated at 30°C for one day. Based on colony shape, size, and color, single colonies of different morphologies were selected and purified on Luria–Bertani (LB) agar (10 g tryptone, 5 g yeast extract, 10 g NaCl, 15 g agar, pH 7.0) by the streaking method. The purified strains were inoculated into liquid LB medium containing 15% glycerol and stored at −80°C.

#### Determination of antimicrobial activity

The bacterial cell suspension (1 × 10⁸ CFU·mL⁻^1^) was prepared following the method of Duan et al. (2021). Antimicrobial activity against plant pathogens was evaluated using the dual culture assay system in PDA refer to the method of Li et al. (2010) and Duan et al. (2021).

#### Inhibition of *Fusarium* growth by LRB-5

The LRB-5 fermentation broth (FB) and cell-free culture filtrate (CFCF) were prepared according to the method described by Duan et al. (2021) and Azabou et al. ( 2020). The effect of CFCF on *Fusarium* (*F. oxysporum* and *F. solani*) hyphal growth and spore germination (*F. oxysporum*, *Fpmd* MR5, *F. verticillioides*, *F. proliferatum*, and *F. solani*) was assessed following the methodology of Duan et al. (2021).

### Identification of biocontrol bacteria

The morphological identification was conducted based on the methods of Duan et al. (2021). Physiological and biochemical characteristics were assessed using the methods described in *Bergey’s Manual of Systematic Bacteriology* (2nd edition) and the *Common Bacterial Identification Manual* (Garrity 2007; Vos et al. 2011). The GEN III MicroPlateTM test panel identification was based on the methods of Duan et al. (2021).

Genomic DNA was extracted using the EasyPure Bacteria Genomic DNA Kit (TransGen Biotech, Beijing, China). To confirm the species identity of LRB-5, we obtained the DNA sequences of the 16S ribosomal RNA gene (16S rRNA), DNA gyrase subunit A (*gyrA*), DNA gyrase subunit B (*gyrB*), and RNA polymerase subunit B (*rpoB*). Polymerase chain reaction (PCR) amplification, product purification, and sequencing refer to the method of Duan et al. (2022a). The primers and annealing temperatures are listed in Table S1.

For phylogenetic analysis, 16S rRNA, *gyrA*, *gyrB*, and *rpoB* sequences were aligned using maximum likelihood (ML) methods were performed for the datasets using RAxML-HPC2 on XSEDE (8.2.12) on the CIPRES website (http://www.phylo.org). Tree diagrams were created using FigureTree v1.4.3 and Adobe Illustrator CS6 (Duan et al. 2022a,b).

### Antagonistic effects of volatile organic compounds on pathogen growth

The antagonistic effects of Volatile organic compounds (VOCs) produced by LRB-5 were measured according to the methods described by Duan et al. (2022a).

### Inhibition of mycelial growth by CFCF

The acquisition of CFCF was as described above. CFCF was added to a warm PDA medium (55°C) to final concentrations (ranging from 0.1% to 1.5%). PDA plates without culture filtrate were used as controls. The plant pathogen mycelial plugs of 10 mm diameter were placed centrally on the amended media and incubated at 25°C until the negative control growth covered the entire surface of the plate. Inhibition of the pathogen growth was estimated using the formula described by Duan et al. (2021).

### Plant growth promoting activities

The ability of strain LRB-5 to promote plant growth was tested according to the method described by Duan et al. (2022b), which mainly evaluated plant growth promoting (PGP) properties (phosphate solubilization, potassium solubilization, nitrogen fixation, ferric siderophore production, ammonia production, amylase production), cell wall-degrading enzyme activity (cellulose, pectinase, β−1,3-glucanase, chitosanase, protease activity). The free amino acids and indole-3-acetic acid (IAA) contents in the FB of strain LRB-5 were determined using a High performance liquid chromatography-tandem mass spectrometry (HPLC–MS/MS). The analysis was performed by Nanjing Ruiyuan Biotechnology Co., Ltd. (Nanjing, China).

### Plant growth promotion by VOCs and FB

The growth promotion of *Arabidopsis thaliana* Col-0 by exposure to LRB-5 was measured according to the method described by Duan et al. (2022a). The acquisition of FB was as described above. FB was centrifuged at 4000 g for 10 min and then redissolved in 10 mM magnesium sulfate solution, with OD_600_ adjusted to 0.5. Ten microliters of the solution were dropped onto the root tips of *Arabidopsis thaliana*, using sterile distilled water as a control. Each treatment was replicated three times. Plant fresh weight, primary root length, and lateral root number were measured after 7 days of incubation.

### Identification of VOCs

The VOCs produced by LRB-5 were determined using a solid-phase micro-Gas chromatography-mass spectrometry (SPME‐GC‐MS) according to the method described by Duan et al. (2022a) and Wu et al. (2019). The mass spectral data for the volatile compounds were analyzed using data from the NIST23 Mass Spectrum Library. The peak area normalization method was used to calculate the relative content of each component.

### Verification of the effects of synthetic compounds on pathogens

Nine standard compounds identified among the VOCs were purchased from a reagent company (Table S2). The antimicrobial activity of the standard compounds was assessed using the I-plate system described by Duan et al. (2022a). The I-plate was prepared with PDA on one side and inoculated with a 5-mm pathogen plug. Then, the nine synthetic compounds were diluted separately in ethanol or distilled water, and 20 μL of the resulting suspension was applied to a sterile filter paper disk on the other side of the I-plate. A concentration of 1000 μg·L⁻^1^ of each synthetic compound was tested. The I-plate was sealed with Parafilm and incubated at 28°C. I-plates with distilled water were used as controls. The colony diameters of the pathogens were recorded after 7 days of incubation, and the experiment was repeated three times.

### Plant growth promotion activities of synthetic compounds

In brief, three 2-day-old germinated *A. thaliana* Col-0 seedlings were transferred to one side of an I-plate containing 0.5 × MS, 0.8% sucrose, and 1% Bacto agar. Then, the synthetic compounds were diluted separately in sterile water, and 20 μL of the resulting suspension was applied to a sterile filter paper disk on the other side of the I-plate. Multiple dosages (10, 100, 500, and 1000 μg) of each synthetic compound were tested, and each treatment was repeated three times. The fresh weights of the seedlings were measured after 10 days.

### Disease assessment

The technique for plant inoculation was based on López-Escudero et al. (2004). Plants were inoculated by dipping their bare root systems in a conidial suspension of *Fusarium* species (10⁶ conidia·mL⁻^1^) for 30 min as positive controls. They were then transplanted into AC140 pots containing a soil mixture (vermiculite/soil, 2:3, v/v) and inoculated with fermentation broth (1 × 10⁸ CFU·mL⁻^1^) by the root irrigation method to serve as the experimental group. Plants not inoculated with *Fusarium* and then drenched with LRB-5 served as the negative controls. Plants were grown under a 16 h light/8 h dark photoperiod at 28°C, and they were watered as required for plant growth and disease development. Each pot contained one *M. hupehensis* Rehd. seedling, and all pots were arranged randomly with three replicates per treatment and 30 pots per replicate. Disease severity was estimated over the course of 5 weeks, starting 1 week after inoculation. Scoring of wilting symptoms on a 0–4 scale was performed using the criteria developed by Azabou et al. (2020). The percentage of dead plants (PDP) was calculated to estimate wilt severity and the ability of plants in different treatment groups to recover from the disease. The area under the disease progress curve (AUDPC) (López-Escudero et al. 2004) for each treatment was assessed according to the formula AUDPC = [(t/2 × (S₂ + 2 × S₃ + ··· + 2S_i_₋₁ + S_i_))/4 × n] × 100, where *t* is the interval between observations in days, *S*_*i*_ is the final mean severity (disease index), 4 is the maximum disease rating, and *n* is the number of observations. The final mean severity of symptoms (FMS) was calculated as FMS = ∑(N_i_ × X_i_)/n_i_, where *N*_*i*_ is the number of plants with symptoms, *X*_*i*_ is the value of the symptom score, and *n*_*i*_ is the number of diseased plants. Disease intensity (DI) based on wilting symptoms was calculated using the equation DI (%) = 100 × ∑(N_i_ × X_i_)/(30 × 4), where *N*_*i*_ is the number of plants with symptoms, *X*_*i*_ is the value of the symptom score, 30 is the total number of plants, and 4 is the maximum disease rating. The relative control effect (%) = (DI_CK_ − DI_T_)/DI_CK_ × 100. Disease incidence (%) = number of diseased plants/total number of plants × 100 (Wu et al. 2019). To fulfill Koch’s postulates, the fungi were re-isolated from discolored fibrous roots of seedlings. Isolation and identification of each organism were performed to the genus level.

### The protective effect of LRB-5 on plant roots

The protective function of strain LRB-5 on the roots of *M. hupehensis* Rehd. seedlings was verified by Periodic Acid Schiff (PAS) staining according to the method of Duan et al. (2022b).

### Field experimental materials

Soil was collected from a 31-year-old apple orchard in Manzhuang Town, Taian, China (117.081039 longitude, 36.06682 latitude). The soil samples were collected 80 cm from the trunk and 20–40 cm from the soil surface using the five-point sampling method. The physicochemical properties of the tested soil are presented in Table S3.

Microbial fertilizer was produced by Chuangdi Microbial Resources Co., Ltd. (Dezhou, China). The bacterial manure carrier consisted of cow dung and straw at a ratio of 3:1, and the bacterial density was 2.1 × 10⁹ CFU·g⁻^1^. The content of available nitrogen was 0.37 mg·g⁻^1^, available phosphorus was 1.46 mg·kg⁻^1^, and available potassium was 1.01 mg·kg⁻^1^.

### Pot experiment

The pot experiment was conducted at the National Apple Engineering Experiment Center of the Horticultural Science and Engineering College of Shandong Agricultural University and the State Key Laboratory of Crop Biology (117.156540°N, 36.164443°E latitude). In May 2017, *M. hupehensis* Rehd. seedlings were transplanted into clay pots (38 cm × 28 cm × 26 cm) containing 75.43 kg of the soil. There were two seedlings per pot and 20 pots per treatment. Potted soil was divided into the following four treatments: 31-year-old orchard soil (CK1), 31-year-old orchard soil fumigated with methyl bromide (CK2), bacterial fertilizer carrier (T1), and LRB-5 bacterial fertilizer (T2). The application amount of bacterial fertilizer and manure carrier accounted for about 1% of the soil weight (Duan et al. 2022b). All indexes were measured on July 15, August 15, and September 15, 2017. The sample collection and management were conducted in accordance with that done by Duan et al. (2021).

#### Measurement indices

##### Microbial culture methods

Soil microbial populations (bacteria, fungi, and actinomycetes) were assessed using the dilution method of plate counting described in Duan et al. (2022a).

##### Plant growth measurements

The plant height and basal diameter of *M. hupehensis* Rehd. seedlings were measured with a measuring stick and a vernier caliper (Sangon Biotech, Shanghai), and the dry and fresh weights were measured with an electronic balance (OLABO, China). An Epson Perfection V850 Pro scanner (Epson [China] Co., Ltd.) was used to scan the root systems. The Wanshen LA-S series plant root analysis system was used to analyze and process the sample images and record the total root length, surface area, forks, and tips.

##### Quantitative determination of soil phenolic acids by HPLC

Soil phenolic acid content was measured using the method described by Duan et al. (2022b).

##### Root antioxidant and soil enzyme activities

The root tissues samples from all the treatments were placed in a container filled with ice for testing. Root tips or white roots were selected to determine the activities of antioxidant enzymes. The activities of superoxide dismutase (SOD), peroxidase (POD), catalase (CAT), and the content of malondialdehyde (MDA) in the root system, as well as the activities of solid-urease (S-UE), soil acid phosphatase (S-ACP), solid-sucrase (S-SC), and solid-catalase (S-CAT) in the soil, were determined using corresponding assay kits from Suzhou Keming Biotechnology Co., Ltd. (Suzhou, China).

##### Quantitative PCR analysis

For each replicate pot described above, a 5-g rhizosphere soil sample was obtained, and DNA was extracted using the PowerMax Soil DNA Isolation Kit (Qiagen, Hilden, Germany). The quality and quantity of DNA were determined using an Eppendorf BioPhotometer nucleic acid and protein analyzer (Eppendorf, Hamburg, Germany). Quantitative PCR (qPCR) was used for the quantification of total bacteria and fungi as well as *Fusarium* and the biocontrol agent LRB-5 (*B. vallismortis*) in the rhizosphere soil samples. Abundances of bacteria and fungi were determined with Eub338F/Eub518R and ITS1f/5.8 s primers, respectively, following established protocols (Fu et al. 2017) using the CFX96 Touch Real-Time PCR Detection System (Bio-Rad, Hercules, CA, USA). Standard curves were generated using tenfold serial dilutions of a plasmid containing a full-length copy of the 16S rRNA gene from *Escherichia coli* and the 18S rRNA gene from *Saccharomyces cerevisiae*. The abundance of *Fusarium* was determined using the SYBR Green assay with the primers JR/JF, CHR/CHF, CR/CF, FR/FF, and MR5R/MR5F (Duan et al. 2022c; Table S1), which target the rRNA internal transcribed spacer (ITS). For the biocontrol agent LRB-5, group-specific primers BaF and BaR (Table S1) were designed for the qPCR assay based on a specific region by comparison with other *Bacillus* genomes in GenBank. The standard curve was generated using a tenfold dilution series of plasmid DNA containing a fragment of the genome from *B. vallismortis*. The primers and annealing temperatures are presented in Table S1. Melting curve analysis and gel electrophoresis were performed to confirm amplification specificity. Gene copy numbers of the target group for each reaction were calculated from the standard curves. Each sample was analyzed in triplicate, and the results are expressed as log_10_ values (target copy number g⁻^1^ soil).

##### Biolog plate analysis

Biolog-ECO plates (Biolog, Hayward, CA, USA) and a plate reader (Multiskan MK3 ELISA) were used to determine the community-level physiological profiles, with specific methods referenced in Duan et al. (2022b). The OD value at 96 h of incubation was used for subsequent categorized substrate utilization pattern analysis.

##### Terminal restriction fragment length polymorphism analysis

Terminal restriction fragment length polymorphism (T-RFLP) analysis was used as a tool to rapidly and qualitatively compare fungal and bacterial community structures across the different treatments, with specific methods referenced in Duan et al. (2022b). DNA was amplified using the universal primers 27F-FAM/1492R and ITS1F-FAM/ITS4R (Table S1), which target the bacterial variable region of the 16S rRNA gene and the fungal ITS region between the 18S and 28S rRNA regions, respectively. The forward primers were labeled at the 5′ end with 6-carboxyfluorescein (FAM), synthesized by Sangon Biotech (Shanghai, China). All PCR amplifications were performed on an Applied Biosystems 2720 Thermal Cycler (Applied Biosystems Inc., USA). PCR products were cleaned with the EZNA PCR Purification Kit (Omega Bio-Tek, USA) following the manufacturer's instructions and quantified using a DNAmaster nucleic acid and protein analyzer (Dynamica, United Kingdom). The purified PCR product (500 ng) was digested by the restriction endonuclease MspI (TaKaRa, Japan) for 16S rRNA gene amplicons and HinfI (TaKaRa, Japan) for ITS amplicons in two separate reactions and sequenced by Sangon Biotech Co., Ltd. (Shanghai, China).

### Data analysis

The experimental data are presented as the mean and standard deviation (SD) of three biological replicates. The effects of treatments were determined by analysis of variance (ANOVA) using IBM SPSS 20.0 (IBM SPSS Statistics, IBM Corporation, Armonk, NY, USA), and significance differences between treatment means were determined using Duncan’s multiple range test at a threshold of *P* < 0.05. The figures were plotted using Microsoft Excel 2013 (Microsoft Corporation, Redmond, Washington, USA) and GraphPad Prism 8.0.2 (GraphPad Software, Inc., San Diego, California, USA). T-RFLP profiles were analyzed using Peak Scanner Software v1.0 (Thermo Fisher Scientific, Wilmington, USA), with detailed methods as described in Duan et al. (2022a). The Ouyi cloud analysis platform (https://cloud.oebiotech.cn/) was used for principal component analysis (PCoA) and cluster analysis to study differences in community composition among samples.

## Supplementary Information

Supplementary Material 1.

Supplementary Material 2.

## Data Availability

The datasets generated and analyzed during the current study are available from the corresponding author on reasonable request. The datasets relevant to this study and presented here can be found in the following online repositories. Details of the repository (ies) and the corresponding accession number (s) are listed below: https://www.ncbi.nlm.nih.gov/genbank/, MT713121.1 (*rpoB*), MT713125.1 (*gyrA*), MN726441.1 (16S rRNA) and MT713120.1 (*gyrB*). LRB-5 was deposited at China General Microbiological Culture Collection Center (CGMCC) under the accession number CGMCC No. 15306.

## Abbreviations

*F. solani*: *Fusarium solani*
*F. oxysporum*: *Fusarium oxysporum*
*F. proliferatum*: *Fusarium proliferatum*
*F. **verticillioides*: *Fusarium* *verticillioides*
*Fpmd* MR5: *Fusarium proliferatum* MR5 f.sp. *m**alus domestica*
*B. vallismortis*: *Bacillus vallismortis*
*A. alternata*: *Alternaria alternata*
*A. verrucaria*: *Albifimbria verrucaria*
*A. flavus*: *Aspergillus flavus*
*P. macrostoma*: *Phoma macrostoma*
*P. brasilianum*: *Penicillium brasilianum*
*R. solani*: *Rhizoctonia solani*
*V. ** mali*: *Valsa mali*
LB: Luria–Bertani
FB: Fermentation broth
PDA: Potato dextrose agar
TSA: Trypticase Soy Agar
16S rRNA: 16S ribosomal RNA gene
*gyrA*: DNA gyrase subunit A
*gyrB*: DNA gyrase subunit B
*rpoB*: RNA polymerase subunit B
ML: Maximum likelihood
VOCs: Volatile organic compounds
SPME: Solid-phase micro-extraction
GC–MS: Gas chromatography-mass spectrometry
PDP: The percentage of dead plants
AUDPC: The area under the disease progress curve
FMS: The final mean severity of symptoms
DI: Disease intensity
*M**. ** hupehensis* Rehd. seedlings: *Malus hupehensis* Rehd. seedlings
ARD: Apple replant disease
qPCR: Real-time quantitative PCR
T-RFLP: Terminal-restriction fragment length polymorphism
SOD: Superoxide dismutase
POD: Peroxidase
CAT: Catalase
MDA: Malondialdehyde
S-UE: Solid-urease
S-ACP: Soil acid phosphatase
S-SC: Solid-sucrase
S-CAT: Solid-catalase
PCoA: Principal coordinate analysis
ITS: Internal Transcribed Spacer
SD: Standard deviation
ANOVA: Analysis of Variance
CGMCC: China General Microbiological Culture Collection Center
IAA: Indole-3-acetic acid
AWCD: Average Well Color Development
ACC: 1-Aminocyclopropane-1-carboxylic acid
PGP: Plant growth promoting
RI: Retention index
SI: Similarity index
TIC: Total ion chromatogram
