# Supplementary material for: Bacillus vallismortis LRB-5: a promising biocontrol agent for mitigating apple replant disease through pathogen suppression and growth promotion
Source: Stress Biol. 2025 Aug 25;5(1):51. doi: 10.1007/s44154-025-00246-5 (PMC12375528; doi:10.1007/s44154-025-00246-5)
Supplement: Supplementary file 1 — Supplementary Material 1. [file 44154_2025_246_MOESM1_ESM.docx]

**Supplementary material**

**Fig. S1** Replant disease-like symptoms (F-J) observed in localized areas within orchards, while the rest of the orchard exhibited uniform growth and standard production (A-E). Replant disease-like symptoms included reduced plant vigor (characterized by lower yield, poorer fruit quality compared to the orchard standard, and leaf chlorosis) or plant death.

**
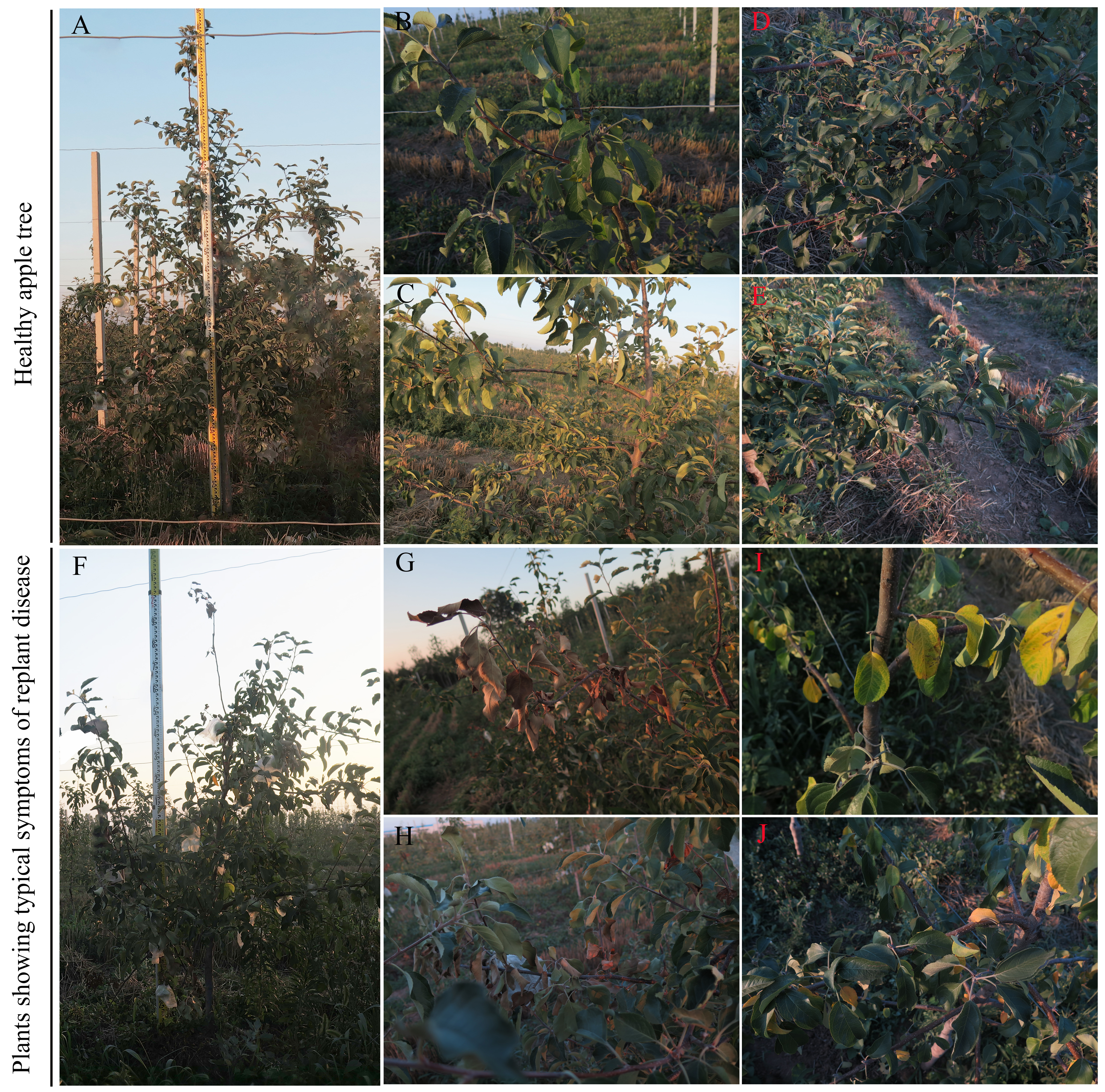
**

**Fig. S2** Antifungal activity of LRB-5 against plant pathogens after 7 d of incubation at 28°C. The center of the PDA medium contained a 1.0 cm diameter mycelial disk, and four sterilized filter papers (6 mm diameter) were spotted with 5 μL of strain LRB-5 cell suspension (1 × 10⁸ CFU·mL⁻¹). Alternatively, LRB-5 was streaked on four extremes of the plate, forming a square around the pathogenic fungi. After culturing Penicillium brasilianum on the PDA plate for 7 d, the PDA plate was rinsed with sterile distilled water. The density of the conidial suspension was measured using a hemocytometer, and the inoculated spore culture was diluted with sterile distilled water to obtain a final concentration of 1 × 10⁶ spores·mL⁻¹. The conidial suspension was added to the PDA medium, mixed, and the plate was inverted before performing the antibacterial test. **A1-2:** Fusarium proliferatum, **B1-2:** Fusarium verticillioides, **C1-2:** Fusarium solani, **D1-2:** Fusarium oxysporum, **E:** Alternaria alternata, **F:** Rhizoctonia solani, **G:** Valsa mali, **H:** Phoma macrostoma, **I:** Aspergillus flavus, **J:** Albifimbria verrucaria, **K:** Penicillium brasilianum.

**
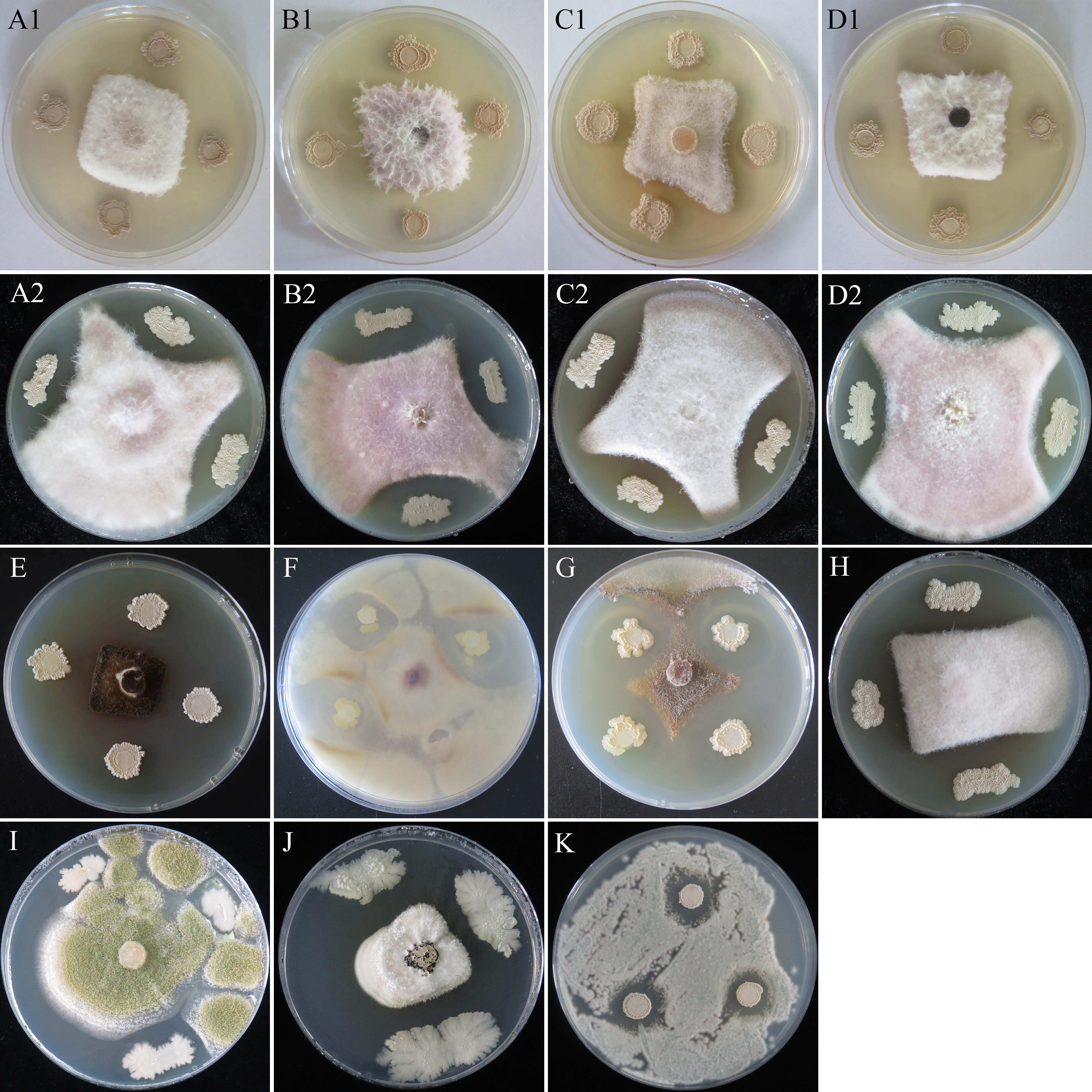
**

**Fig. S3** The isolated LRB-5 was cultured on LB agar at 37°C for 24 h (A), and the cellular morphological characteristics were observed using a Nikon microscope BX-51 (100×/1.30 oil lens) (B-C) and a scanning electron microscope SU-8010 (D-I). B: The endospore staining of LRB-5 using malachite green, bar = 6 μm. C: The staining of LRB-5 using Gram staining, bar = 5 μm. D-H: bar = 10 μm. G-I: bar = 2 μm.


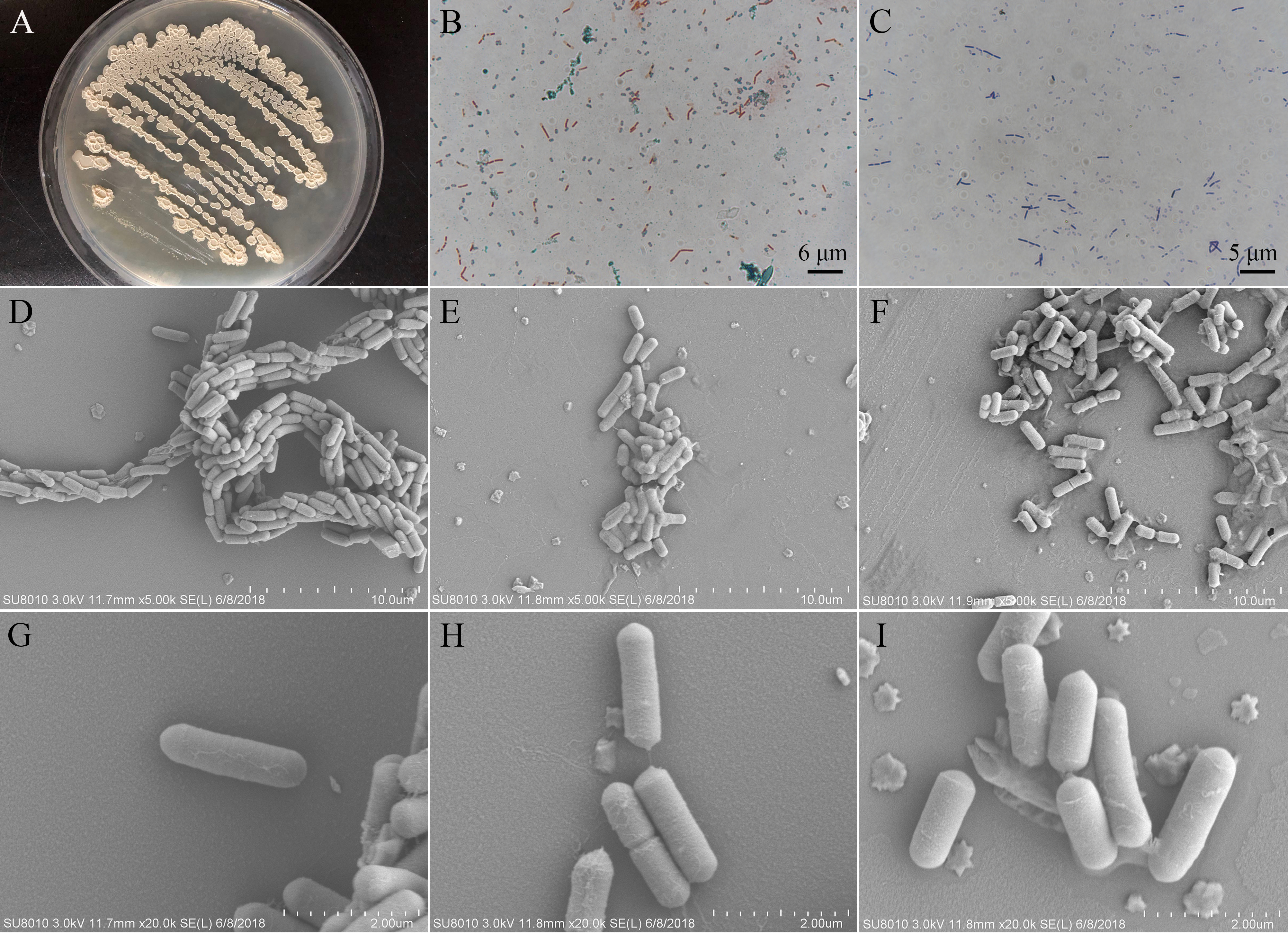


**Fig. S4** The ML consensus tree inferred from the combined 16S rDNA, gyrA, gyrB, and rpoB sequence alignment. Support for each branch in the inferred tree was evaluated using 1000 bootstrap replications. Support values (ML bootstrap and posterior probability values) are indicated at the branches. The scale bar indicates 0.2 expected changes per site. Clade numbers and Latin names are provided on the right of the tree and are used for reference in the treatment of the species. The tree is rooted to Paenibacillus polymyxa (BLB267). LRB-5 is indicated in bold and red.





**Fig. S5** Effect of cell-free culture filtrate on mycelia of *Fusarium solani* . A-D were the blank control mycelium, E-H were the mycelium treated with cell-free culture filtrate. Bar = 20 μm.

**
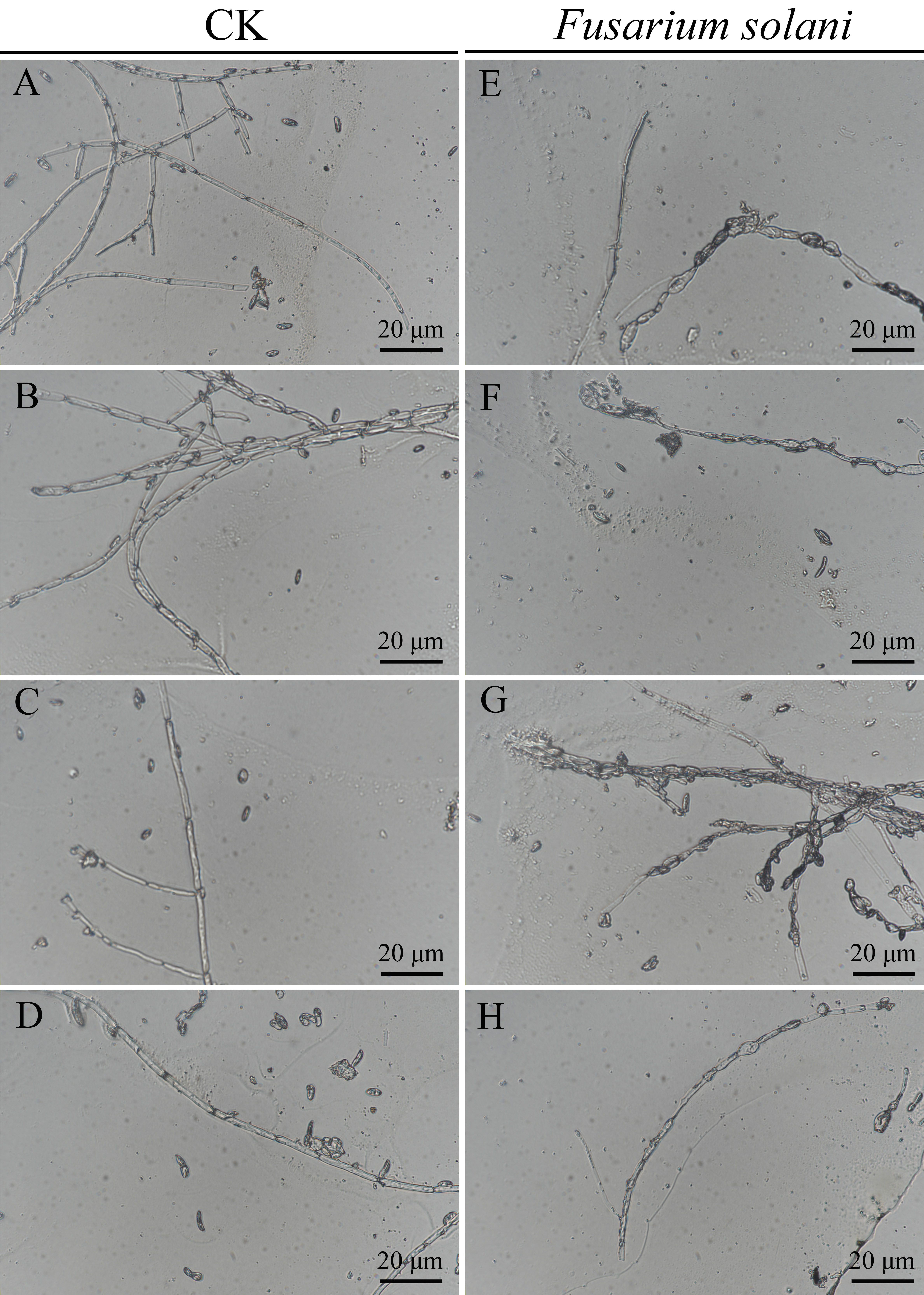
**

**Fig. S6** The mycelium and spore morphology of *Fusarium oxysporum* under the scanning electron microscope. A-C: Normal growing mycelium and spores. D-H: The mycelium treated with cell-free culture filtrate of LRB-5. The mycelium showed irregular reticulation, uneven thickness, shrinkage (D-H), thinning (D, H), breakage, and cell contents overflowed (D-F). The spore cell wall was deformed (H). Scale bar in C: 2 μm. Scale bars in A, B, H: 5 μm. Scale bars in D, F, G, E: 10 μm.


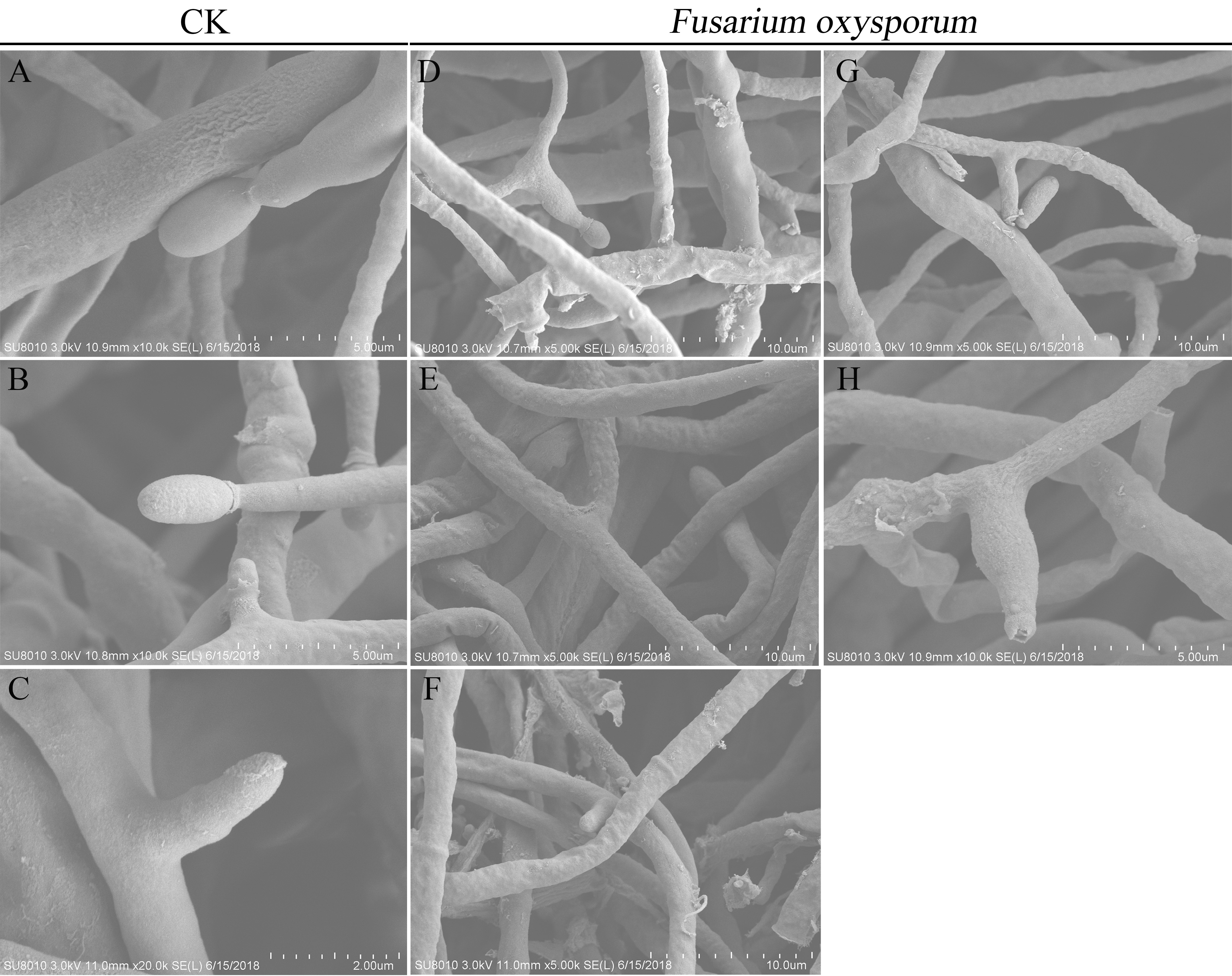


**Fig. S7** Antagonistic effects of LRB-5 on conidia germination of *Fusarium* spp. CK1: *Fusarium* spore suspension was mixed with sterile water at 1:1, CK2: *Fusarium* spore suspension was mixed with fermentation broth at 1:1 (*Bacillus* without antibacterial effect), LRB-5: *Fusarium* spore suspension was mixed with fermentation broth at 1:1, CFCF: *Fusarium* spore suspension was mixed with cell-free culture filtrate at 1:1. *Fpmd* MR5: *Fusarium proliferatum* f. sp. *Malus domestica* MR5.


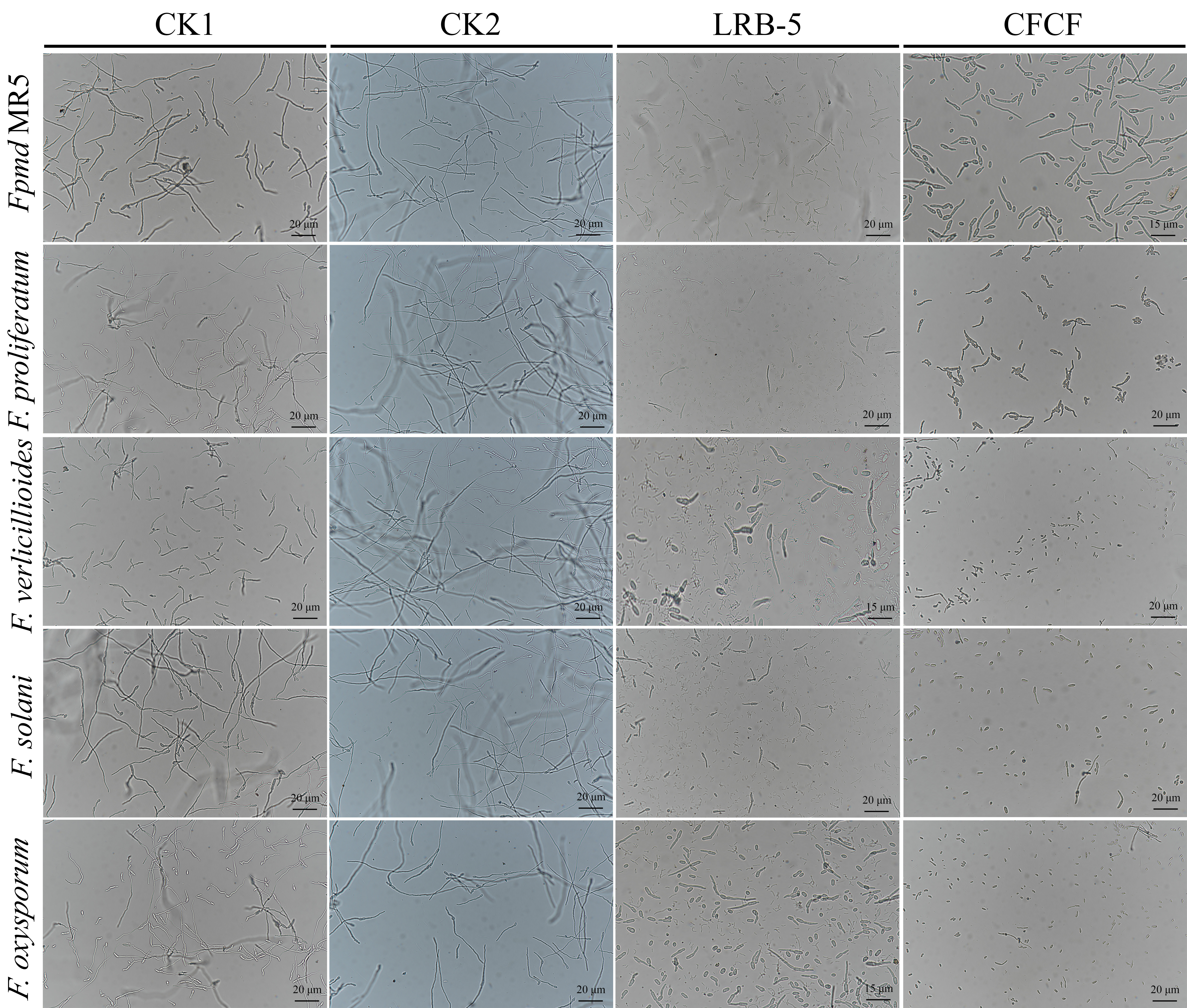


**Fig. S8** The volatile antimicrobial activity of LRB-5 in I-plate and double plate chamber. The control in the I-plate of *Alternaria alternata, Fusarium solani, Albifimbia verrucaria, and Penicillium brasilianum* referred to the study of Duan et al. (2022). A, *Fusarium proliferatum*; B, *Fusarium verticillioides*; C, *Fusarium oxysporum*; D, *Fusarium solani*; E, *Rhizoctonia solani*; F, *Phoma macrostoma*; G, *Penicillium brasilianum*; H, *Aspergillus flavus*; I, *Phytophthora cactorum*; J, *Albifimbria verrucaria*; K, *Alternaria alternata*.

**
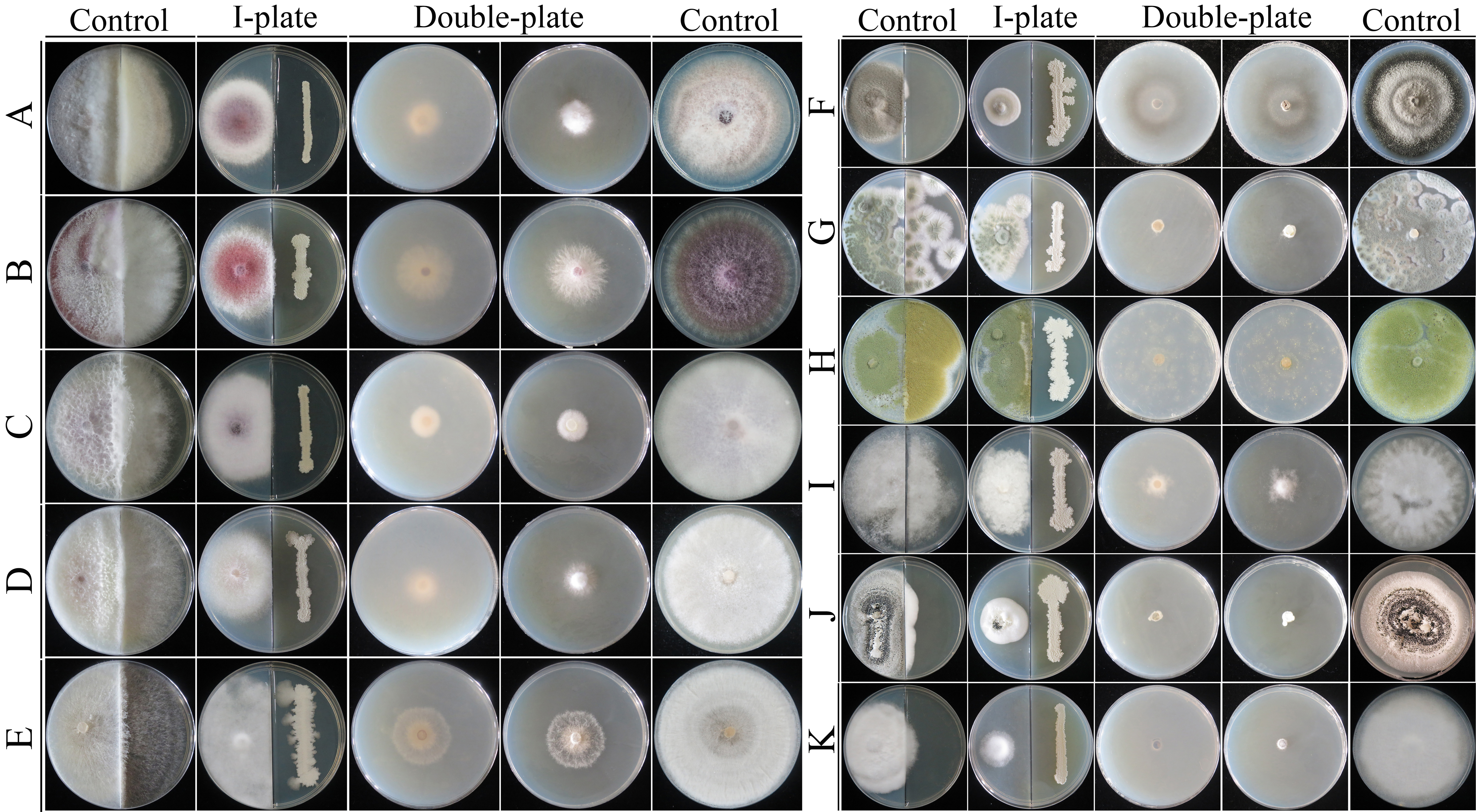
**

**Fig. S9** Total ion chromatogram of LRB-5 volatile antibacterial metabolites extracted by different SPME fibers. The abscissa represents the peak time, and the ordinate represents the peak height. The Fig. is marked with retention time and substance name. Blue: 65 μm PDMS/DVB, Red: 100 μm PDMS, Grey: 50/30 μm PDMS/DVB/CAR.


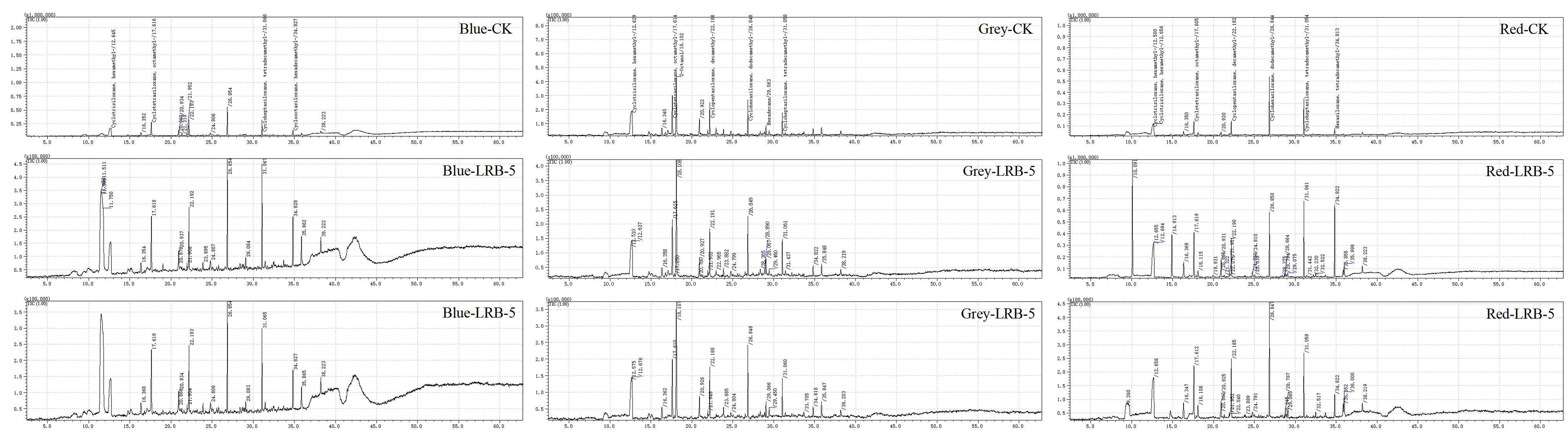


**Fig. S10** The antimicrobial activity of pure VOCs on I-plate. The left side of the picture was marked with the tested pathogenic fungus. Solvents (sterile water) were used as controls, A: 5-methyl-2-heptanone, B: Heptacosane, C: Heneicosane, D: 2,5-Dihydroxybenzaldehyde, E: 1-Tetradecanol, F: 1-Hexadecanol, G: 2-Ethyl-1-hexanol, H: Styrene, I: Toluene. The pure VOCs concentration was 1000 μg·L^-1^. a, *Rhizoctonia solani*; b, *Phytophthora cactorum*; c, *Aspergillus flavus*; d, *Penicillium brasilianum*; e, *Albifimbria verrucaria*; f, *Fusarium proliferatum*; g, *Fusarium verticillioides*; h, *Fusarium oxysporum*; i, *Fusarium solani*; j, *Alternaria alternata*.

**
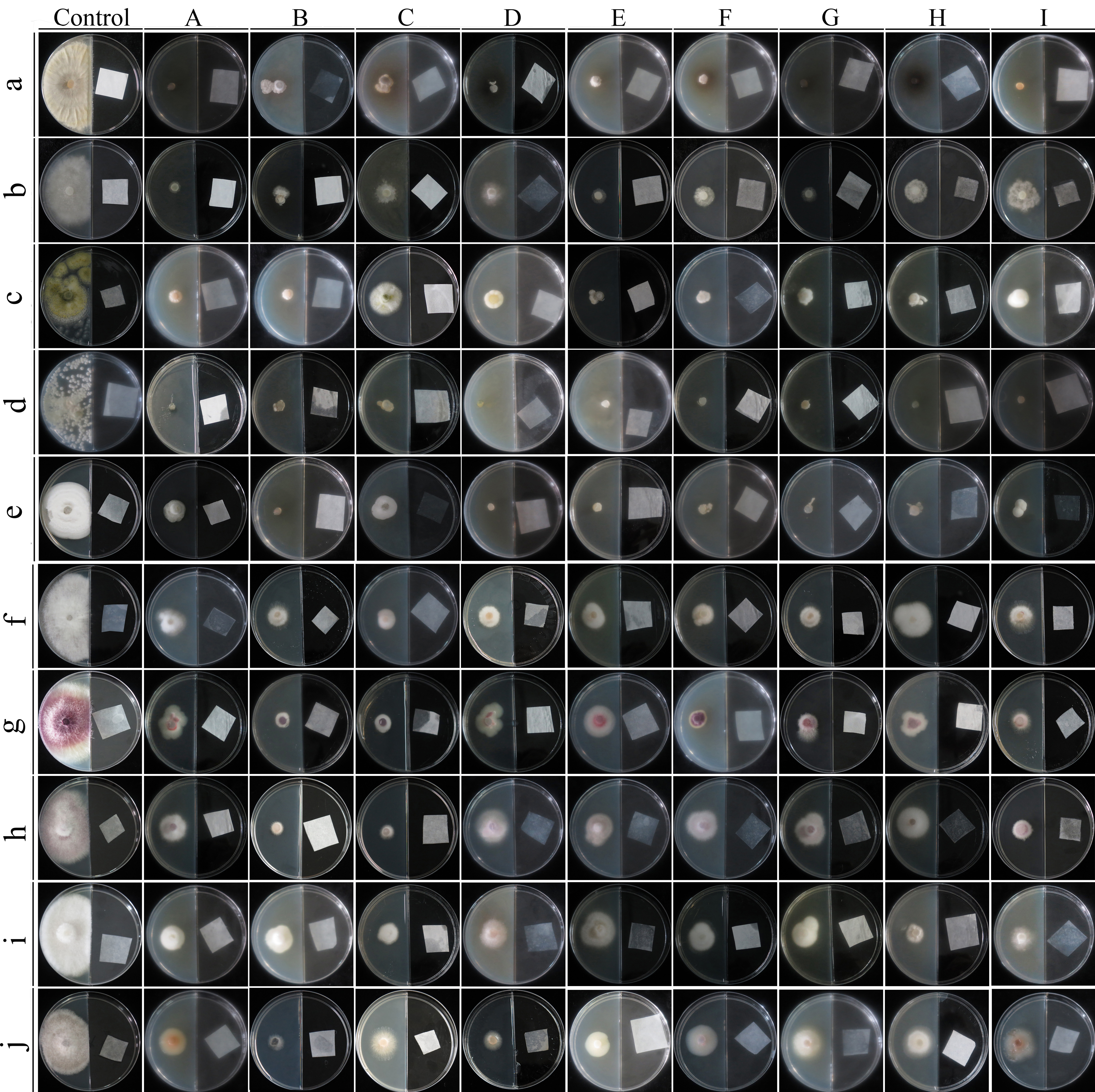
**

**Fig. S11** Strain LRB-5 has a variety of plant growth promoting activities. A: Phosphate solubilization, B: Potassium solubilization, C: Nitrogen fixation, D: Siderophore production, E: Ammonia production, F: Pectinase activity, G: Amylase production, H: Cellulose activity, I: β1,3-glucanase activity, J: Protease activity, K: Chitosanase activity.


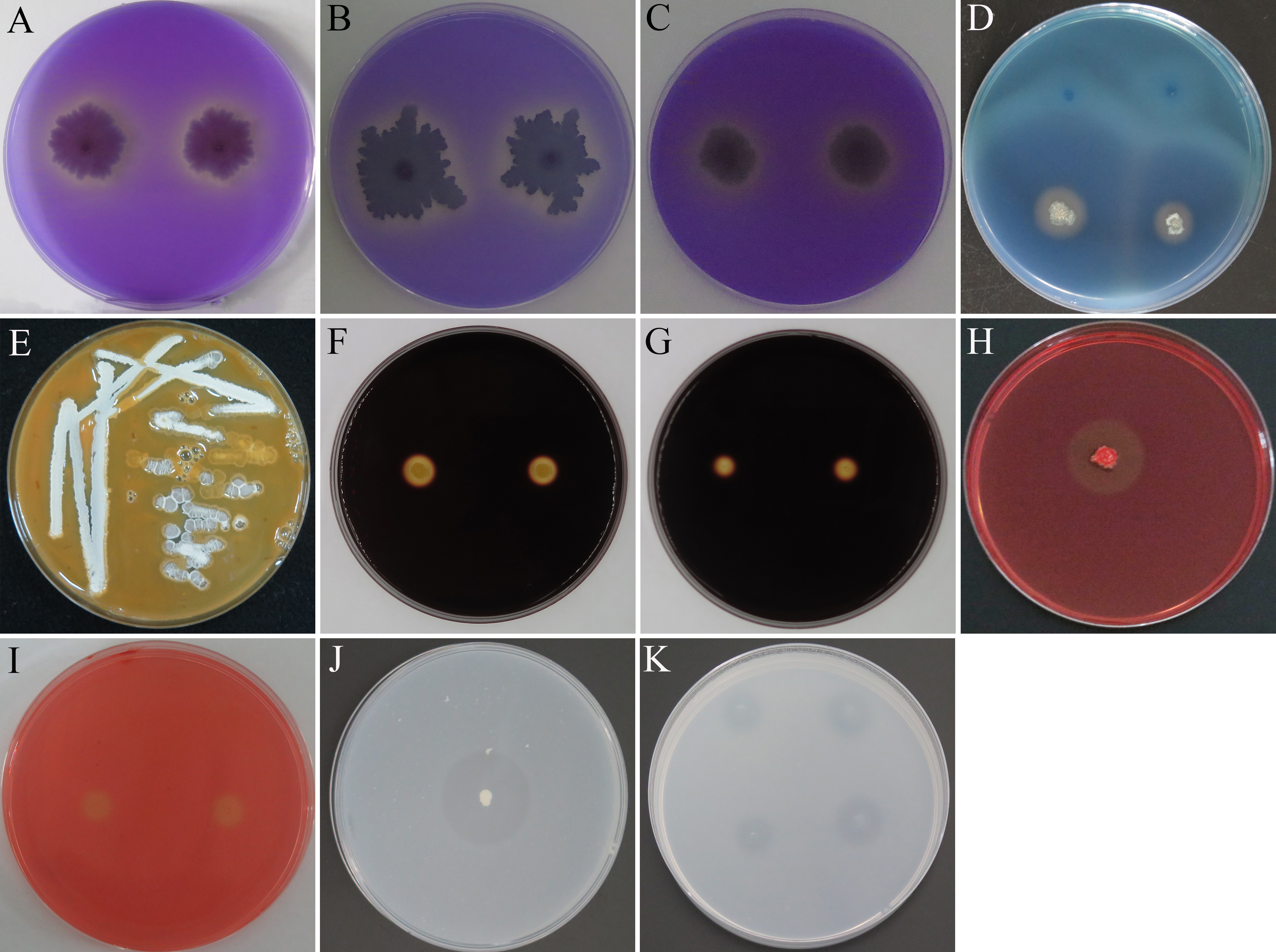


**Fig. S12** Growth promotion of *Arabidopsis thaliana* Col‐0 with exposure to pure VOCs. (a) Wa: Water, A: 2,5-Dihydroxybenzaldehyde (100 μg), B: Styrene (500 μg), C: Heptacosane (100 μg), D: 5-methyl-2-heptanone (100 μg), E: 1-Tetradecanol (100 μg), F: 2-Ethyl-1-hexanol (100 μg), G: 1-Hexadecanol (100 μg), H: Heneicosane (100 μg), I: Toluene (100 μg). J: Plant fresh weight, K: Length of primary root, L: Number of lateral roots. Different lowercase letters above the columns indicate a significant difference at *p* < 0.05. Values are mean±standard deviation (n = 3).

**
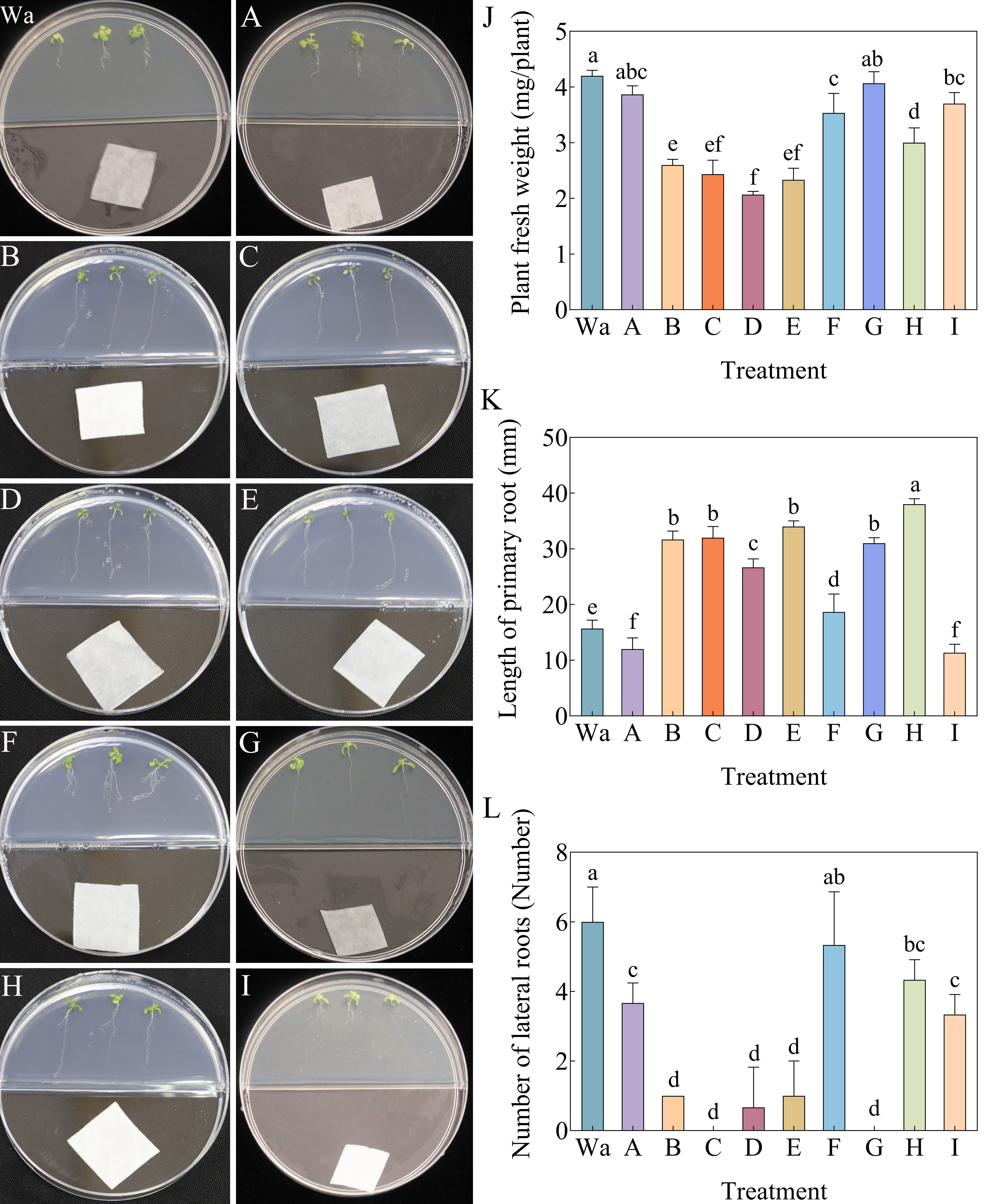
**

**Fig. S13** Symptom severity of *Malus hupehensis* Rehd*.* seedlings from the first to five weeks after inoculation with *Fusarium*. The disease intensity (DI) was measured to estimate wilt severity. Numbers 1, 2, 3, 4, and 5 represent the week. Values are means ±standard deviation of the mean (n = 3).

**

**

**Fig. S14** Microscopic observation of the plant root stained with PAS. Mock: Sterile distilled water control, A-C: the roots were first soaked for 12 h in fermentation broth from LRB-5, then treated with a suspension of *Fusarium oxysporum* spores for 12 h.. D-F: Roots treated with *Fusarium oxysporum*. G-I: Roots treated with *Fusarium solani*. D-I: They soaked the roots of the plants with the conidia suspension for 12h. PAS staining method can dye the polysaccharides on the fungal wall into purple-red. The root system was mainly composed of three parts: (from outside to inside) the epidermis, the cortex (the outer cortex, the cortical parenchyma, the casparian band), and the vascular column (the central sheath, phloem, and xylem). H: Dense mycelium appeared in the epidermis of the root system (arrows). G: Cauliflower-like structure appeared in the infected root areas (arrows). F: Viscous substances and starch granules appear in the cortex and vascular columns (arrows). D, E, I: The conidia and hyphae of *Fusarium* appeared in the cortex and vascular column (arrows).


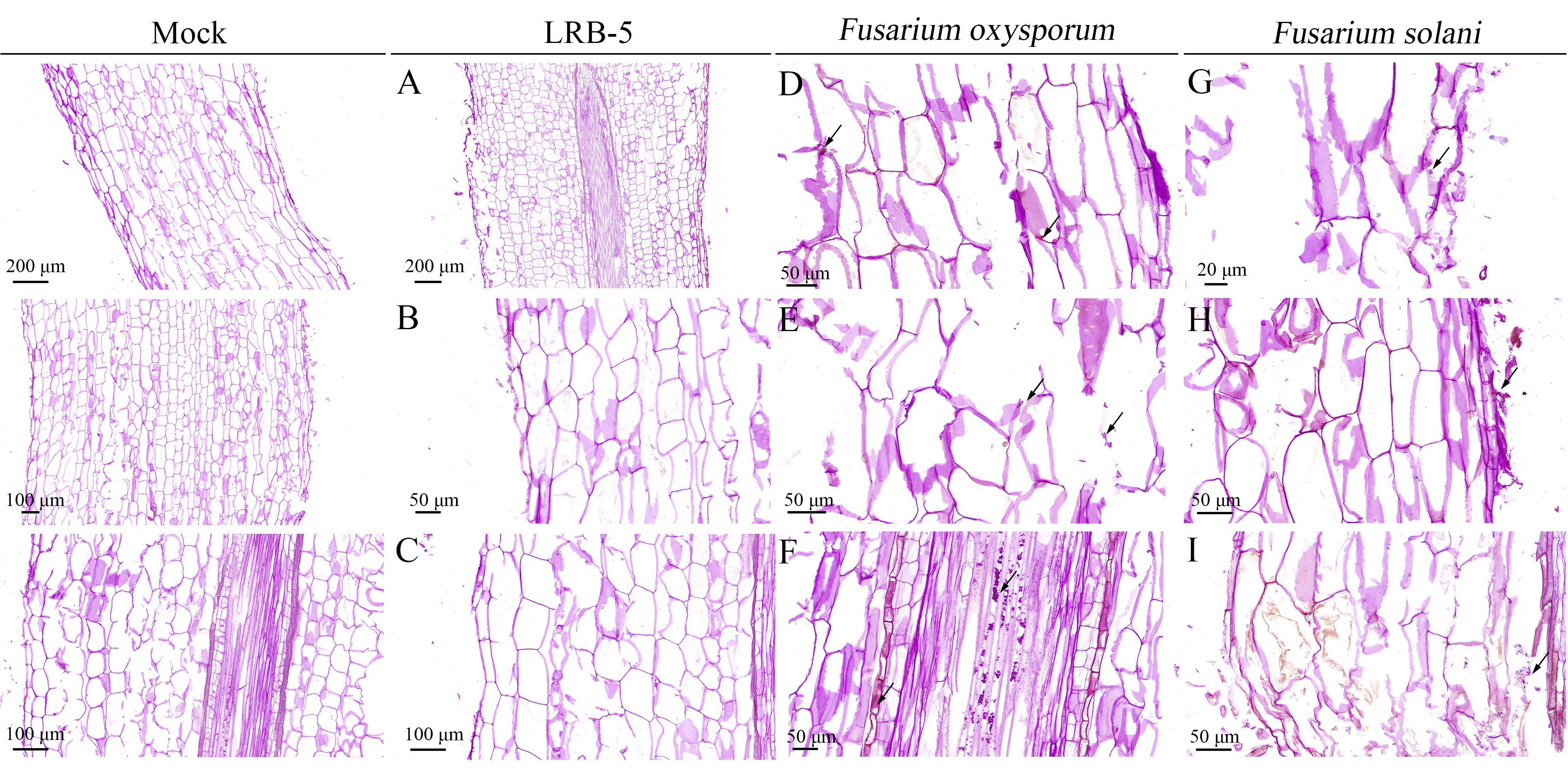


**Fig. S15** Microscopic observation of the plant root stained with PAS. A-C: Roots treated with *Fusarium proliferatum*. D-F: Roots treated with *Fusarium verticillioides*. They soaked the roots of the plants with the conidia suspension for 12h. PAS staining method can dye the polysaccharides on the fungal wall with purple-red color The root system was mainly composed of three parts: (from outside to inside) the epidermis, the cortex (the outer cortex, the cortical parenchyma, the casparian band), and the vascular column (the central sheath, phloem, and xylem). A and D: Viscous substances and starch granules appear in the cortex and vascular columns (arrows). B, C, D, E: Arrow indicates *Fusarium* conidia present in the cortex and vascular cylinder. E and F: Arrow points to a cauliflower-like structure in the infected root zone.


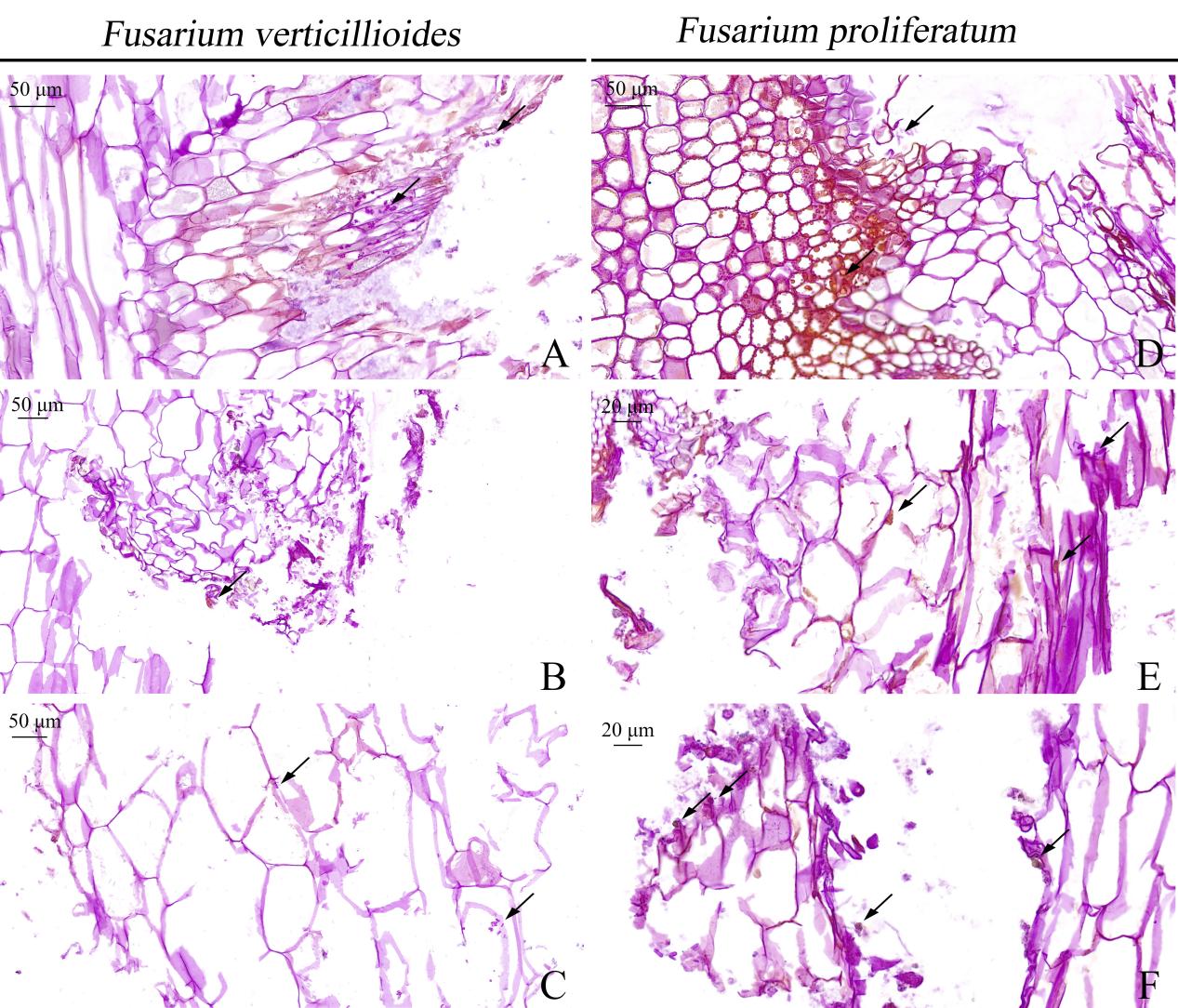


**Fig. S16** Utilization of different carbon substrates by rhizosphere soil samples from September under different treatments using Biolog Eco plates. A: Variation in average well color development (AWCD) after 168 h of incubation. B: Categorized substrates utilization pattern by microbial communities from rhizosphere soils after 96 h of incubation. Values in columns followed by the same letter are not significantly different according to Duncan's test at *p* < 0.05. Values are mean±standard deviation (*n* = 3). CK1: 31-year-old orchard soil, CK2: Methyl bromide fumigation, T1: Fertilizer carrier, T2: LRB-5 bacterial fertilizer.

**
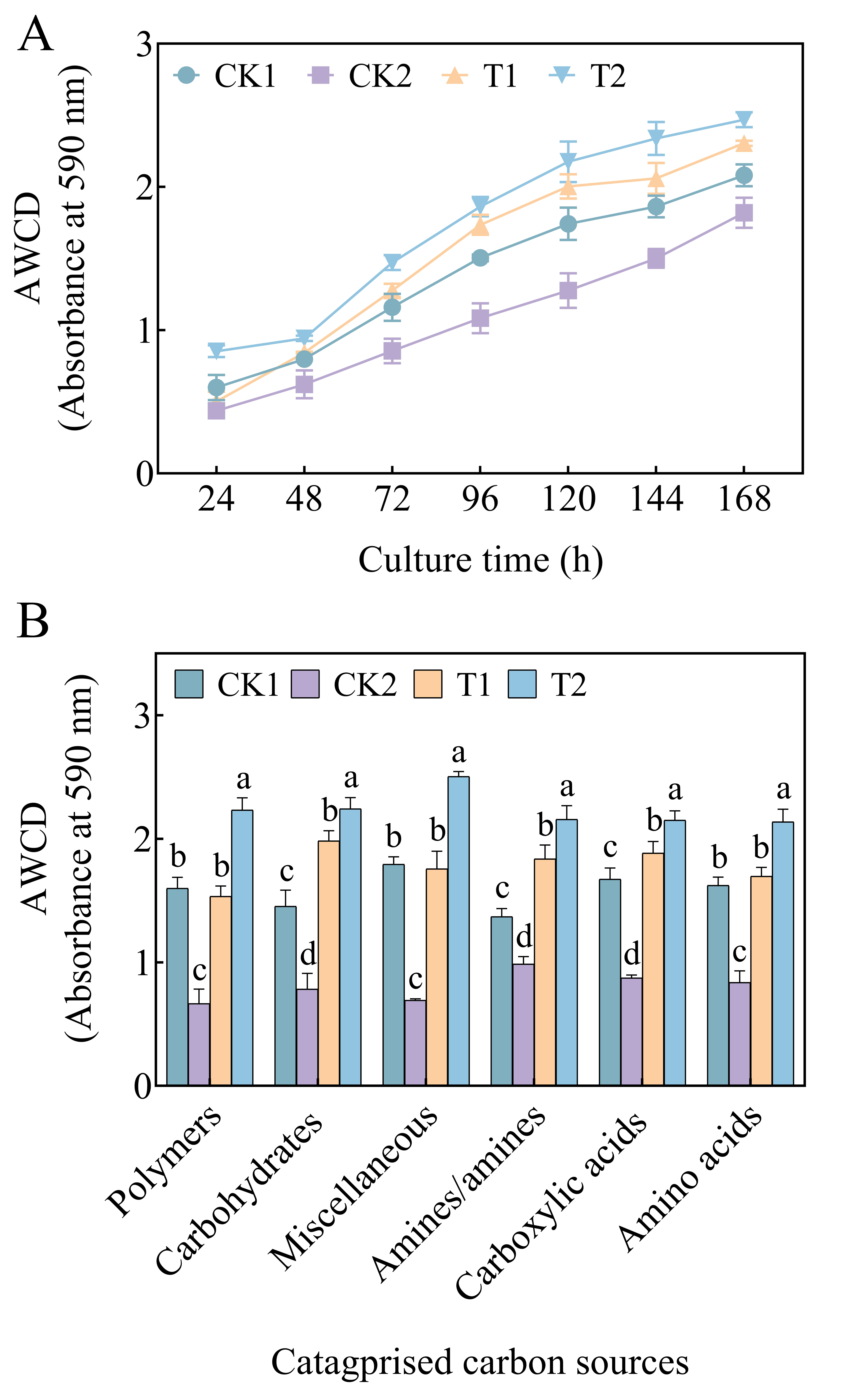
**

**Fig. S17** Effects of different treatments on soil phenolic acids in July, August, and September. A, Cinnamic acid. B, Phlorizin. C, Benzoic acid. D, Ferulic acid. E, *p*-hydroxybenzoic acid. F, Syringic acid. CK1: 31-year-old orchard soil, CK2: Methyl bromide fumigation, T1: Fertilizer carrier, T2: LRB-5 bacterial fertilizer. Values in columns followed by the same letter are not significantly different according to Duncan's test at *p* < 0.05. Values are mean±standard deviation (*n* = 3).

**

**

**References**

Duan Y, Chen R, Zhang R, Jiang W, Chen X, Yin C, Mao Z (2022) Isolation and identification of *Bacillus vallismortis* HSB-2 and its biocontrol potential against apple replant disease. Biological Control 170: 104921.
