# Supplementary material for: Bacillus vallismortis LRB-5: a promising biocontrol agent for mitigating apple replant disease through pathogen suppression and growth promotion
Source: Stress Biol. 2025 Aug 25;5(1):51. doi: 10.1007/s44154-025-00246-5 (PMC12375528; doi:10.1007/s44154-025-00246-5)
Supplement: Supplementary file 2 — Supplementary Material 2. [file 44154_2025_246_MOESM2_ESM.docx]

**Supplementary material**

**Table S1.** Oligonucleotide primers and probes were used in this experiment.

| Primer name | Oligonucleotide sequence (5′–3′) | Gene | Tm°C | Thermal cycling conditions | PCR reaction system | Reference |
| --- | --- | --- | --- | --- | --- | --- |
| 27F/1492R | AGAGTTTGATCCTGGCTCAG  GGTTACCTTGTTACGACTT | 16S rDNA | 57℃ | 4 min at 96°C, followed by 30 amplification cycles of 94°C for 30 s, annealing for 30 s | Mix 2.5 μL of 10x Taq buffer, 1 μL of 50 mM MgCl_2_, 2.5 μL of 2 mM dNTPs, 0.2 μL of Platinum^®^ Taq Polymerase (5 U^.^μL^−1^, Invitrogen™), 5 pmoles of Primer 27F, 10 pmol of primer 1492R and 8 ng of DNA template, and bring them up to a final volume of 25 μL with ultra-pure water | Dos Santos et al. 2019 |
| 42f/1066r | CAGTCAGGAAATGCGTACGTCCTT  CAAGGTAATGCTCCAGGCATTGCT | *gyrA* | 62℃ | 5 min at 94°C; followed by 30 amplification cycles of 95°C for 15 s, annealing for 30 s | Mix 2 μL of the target DNA, 10 μL of 5×PrimeSTAR Buffer (Mg^2+^Plus), 4 μL of dNTP Mixture (2.5 mM each), 1.0 μL of each primer (10 μM), 0.5 μL of PrimeSTAR HS DNA Polymerase (2.5 U^.^uL^-1^), and 31.5 μL sterile distilled water | Chun and Bae 2000 |
| up1f/up2r | GAAGTCATCATGACCGTTCTGCAYGCNGGNGGNAARTTYGA  AGCAGGGTACGGATGTGCGAGCCRTCNACRTCNGCRTCNGTCAT | *gyrB* | 60℃ |  |  | Yamamoto and Harayama 1995 |
| Prpo1/Prpo2 | ATTTCGTTAGCCGAAGAACGT  ATGTGTCCTATTGAGACACCA | *rpoB* | 50℃ |  |  | Zalila-Kolsi et al. 2016 |
| JR/JF | CATACCACTTGTTGTCTCGGC  GAACGCGAATTAACGCGAGTC | *Fusarium oxysporum* | 60℃ | 30 s at 95°C followed by 40 amplification cycles of 94°C for 5 s, annealing for 30 s | Mix 2 μL of the target DNA, 10 μL of SYBR Green premix Ex Taq (TaKaRa, Japan), 0.4 μL of each primer (10 μM), and 7.2 μL sterile distilled water | Duan et al. 2022b |
| CHR/CHF | GACTCGCGAGTCAAATCGCGT  GGGGTTTAACGGCGTGGCC | *Fusarium verticillioides* | 60℃ |  |  |  |
| CR/CF | GATCGGCGAGCCCTTGCGGCAAG  CGCCGCGTACCAGTTGCGAGGGT | *Fusarium proliferatum* | 65℃ |  |  |  |
| FR/FF | CGAGTTATACAACTCATCAACC  GGCCTGAGGGTTGTAATG | *Fusarium solani* | 65℃ |  |  |  |
| MR5R/MR5F | CGATGCCAGAACCAAGAGATCCG  CCGCCAGAGGACCCCTAAACTC | *Fpmd* MR5^a^ | 65℃ |  |  | Y = -3.182x + 14.120, *R^2^* = 0.999 |
| BaR/BaF | ACTTAAGAAACCGCCTGCGA  CCACACTGGGACTGAGACAC | *Bacillus vallismortis* LRB-5 | 60℃ | 30 s at 95°C, followed by 40 amplification cycles of 95°C for 5 s, annealing for 34 s |  | Y = -2.861x + 10.94, *R^2^* = 0.998 |
| Eub338R/Eub338F | ATTACCGCGGCTGCTGG  ACTCCTACGGGAGGCAGCAG | Bacteria | 60℃ |  |  | Fu et al. 2017 |
| 5.8s/ITS1f | CGCTGCGTTCTTCATCG  TCCGTAGGTGAACCTGCGG | Fungi | 60℃ |  |  |  |
| 27F-FAM/1492R | AGAGTTTGATCCTGGCTCAG  GGTTACCTTGTTACGACTT | 16S rDNA | 52℃ | 3 min at 94°C; followed by 30 amplification cycles of 94°C for 45 s, annealing for 45 s | Mix 0.6 μL of 5 U^.^uL^−1^ Ex Taq (TaKaRa, Japan), 5 μL of 10×Ex Taq Buffer, 1 μL of 2.5 mM dNTP mixture, 2 μL of 0.5 mM forward and reverse primers, 12.6 μL of ddH_2_O, and 2.0 μL containing 100 ng of the extracted DNA template | Duan et al. 2022a |
| ITS1F-FAM/ITS4R | CTTGGTCATTTAGAGGAAGTAA  TCCTCCGCTTATTGATAGC | ITS | 50℃ | 5 min at 95°C; followed by 30 amplification cycles of 94°C for 30 s, annealing for 30 s |  |  |

^a^*Fpmd* MR5: *Fusarium proliferatum* f.sp. *malus domestica* MR5.

**Table S2.** The basic information of pure volatile organic compounds.

| Number | Ingredient name | Solvent | Product properties | Purity | Price | Prostitution |
| --- | --- | --- | --- | --- | --- | --- |
| 1 | 1-Tetradecanol | Enthanol | ≥ 99% (GC) | 200 mg | ¥ 100.00 | Shanghai Yuanye Biotechnology Co. , Ltd. |
| 2 | Heptacosane | H_2_O | 98% (GC) | 250 mg | ¥ 349.00 | Shanghai Yuanye Biotechnology Co. , Ltd. |
| 3 | 1-Hexadecanol | H_2_O | 98% (GC) | 25 g | ¥ 220.00 | Shanghai Yuanye Biotechnology Co. , Ltd. |
| 4 | Heneicosane | H_2_O | ≥ 99% (GC) | 10 mg | ¥ 330.00 | Shanghai Yuanye Biotechnology Co. , Ltd. |
| 5 | Toluene | H_2_O | ≥ 99.5% | 500 mL | ¥ 20.00 | Shanghai Titan Scientific Co. , Ltd. |
| 6 | 2,5-Dihydroxybenzaldehyde | H_2_O | 98% | 1 g | ¥ 90.00 | Shanghai Yuanye Biotechnology Co. , Ltd. |
| 7 | Styrene | H_2_O | > 99.5% (GC) | 5 mL | ¥ 49.30 | Shanghai McLin Biochemical Technology Co. , Ltd. |
| 8 | 5-methyl-2-heptanone | H_2_O | 97.0% | 50 mg | ¥ 965.60 | Shanghai McLin Biochemical Technology Co. , Ltd. |
| 9 | 2-Ethyl-1-hexanol | H_2_O | > 99% (GC) | 500 mL | ¥ 51.85 | Shanghai McLin Biochemical Technology Co. , Ltd. |

**Table S3:** The physical and chemical properties of the soil in the old apple orchard. Values are mean±standard deviation (n = 3).

| Location | Ammonium nitrogen  (mg^.^kg^-1^) | Available Phosphorus  (mg^.^kg^-1^) | Available Potassium (mg^.^kg^-1^) | Organic matter (%) | Soil bulk density content (g^.^cm^-3^) | Soil pH | Soil moisture content (%) | Soil texture | R (%)^a^ |
| --- | --- | --- | --- | --- | --- | --- | --- | --- | --- |
| Lutou Town, Longkou City | 25.66±2.81 | 159.12±23.23 | 143.89±29.11 | 1.83±0.55 | 1.15±0.18 | 6.85±0.27 | 13.11±2.70 | Sandy loam | Severe |
| Manzhuang Town | 1.68±0.02 | 10.57±0.37 | 53.13±6.33 | 1.35±0.02 | - | 6.00±0.23 | 15.49±0.42 | Sandy loam | - |
| Reference | Peverill et al. 1999 | | | | Duan et al. 2022b | | | Avery 1973 | Xiang et al. 2021 |

^a^R = 100 × (X_h_ - X_nh_) / X_nh_, where X_h_ and X_nh_ are the dry biomass accumulations for the h and nh soil treatments respectively. We ranked the severity of ARD in the experimental orchards as Severe (%R > 100%), Moderate (%R = 50 to 100%), and Low (%R < 50%).

- : Not measured.

**Table S4.** The sample location was recorded. Bacteria and biocontrol bacteria were separated from different parts of the plant and the rhizosphere soil. The total number of biocontrol bacteria exhibited effective inhibitory activity against *Fusarium proliferatum*, *Fusarium verticillioides*, *Fusarium oxysporum*, *Fusarium solani*, *Alternaria alternata*, *Phoma macrostoma*, *Aspergillus flavus*, *Rhizoctonia solani*, and *Valsa mali*, respectively.

| Province | Shandong | | |
| --- | --- | --- | --- |
| Local site | Lutou Town, Longkou City | | |
| Location numbera^1^ | L | | |
| Habitat | Number of bacteria | Number of biocontrol bacteria | Biological control potential (%)^2^ |
| Soil | 52 | 21 | 40.38 |
| Root | 24 | 10 | 41.67 |
| Stem | 24 | 6 | 25.00 |
| Leaf | 32 | 12 | 37.50 |
| Fruit | 7 | 1 | 14.29 |
| Total | 139 | 52 | 35.97 |

^1^Naming rules: place name abbreviation + habitat (soil, root, stem, leaf, fruit) + microorganism type + number. For example, the first strain of bacteria screened in the soil of Lutou Town, Longkou City, named LSB-1.

^2^Biological control potential (%) = Total number of biocontrol bacteria / Total number of bacteria isolated × 100%

**Table S5.** Physiological and biochemical characteristics of LRB-5.

| Text index | Results | Text index | Results |
| --- | --- | --- | --- |
| Contact enzyme | + | Gelatin hydrolysis enzyme | + |
| Starch hydrolysis enzyme | + | Glucose fermentation reaction | + |
| Nitrate reduction enzyme | + | Methyl red reaction | - |
| Indole enzyme | - | Voges-Proskauer (V-P) reaction reaction | - |
| Citrate enzyme | + | [Malonate](http://dict.youdao.com/search?q=malonate%0D%0A&keyfrom=fanyi.smartResult) reaction | - |
| Hydrogen peroxide reaction | + | Sucrose fermentation reaction | + |

Note: + : positive reaction; - : negative reaction. The experiment was repeated three times.

**Table S6.** Utilization ability of strain LRB-5 on 94 phenotypic tests. The Biolog GEN III MicroPlate analyzes a microorganism in 94 phenotypic tests: 71 carbon source utilization assays (columns 1-9) and 23 chemical sensitivity assays (columns 10-12). All of the wells start out colorless when inoculated. During incubation there is increased respiration in the wells where cells can utilize a carbon source and/or grow. Increased respiration causes reduction of the tetrazolium redox dye, forming a purple color. Negative wells remain colorless, as does the negative control well (A-1) with no carbon source. There is also a positive control well (A-10) used as a reference for the chemical sensitivity assays in columns 10-12. All wells visually resembling the A-1(A-10) well should be scored as “negative” (-) and all wells with a noticeable purple color (greater than well A-1 and A-10) should be scored as “positive” (+). Wells with extremely faint color, or with small purple flecks or clumps are best scored as “borderline” (-/+).

| Columns | Nutrient matrix | Reaction type | Columns | Nutrient matrix | Reaction type | Columns | Nutrient matrix | Reaction type |
| --- | --- | --- | --- | --- | --- | --- | --- | --- |
| A1 | Negative Control | -/+ | C9 | Inosine | + | F5 | D-Glucuronic Acid | -/+ |
| A2 | Dextrin | + | C10 | 1%Sodium Lactate | + | F6 | Glucuronamide | - |
| A3 | D-Maltose | + | C11 | Fusidic Acid | - | F7 | Mucic Acid | + |
| A4 | D-Trehalose | + | C12 | D-Serine | - | F8 | Quinic acid | -/+ |
| A5 | D-Cellobiose | + | D1 | D-Sorbitol | + | F9 | D-Saccharic Acid | + |
| A6 | Gentiobiose | + | D2 | D-Mannitol | + | F10 | Vancomycin | - |
| A7 | Sucrose | + | D3 | D-Arabitol | - | F11 | Tetrazolium Violet | - |
| A8 | Turanose | + | D4 | Myo-Inositol | + | F12 | Tetrazolium Blue | - |
| A9 | Stachyose | - | D5 | Glycerol | + | G1 | p-Hydroxy  -phenylacetic Acid | - |
| A10 | Positive Control | + | D6 | D- Glucose -6-Phosphate | - | G2 | Methyl pyruvate | + |
| A11 | pH6 | + | D7 | D- Fructose -6-Phosphate | + | G3 | D-Lactic Acid Methyl Ester | + |
| A12 | pH5 | + | D8 | D-Aspartic Acid | - | G4 | L-Lactic Acid | + |
| B1 | D-Raffinose | + | D9 | D-Serine | - | G5 | Citric Acid | + |
| B2 | α-D-Lactose | - | D10 | Troleandomycin | - | G6 | α-Keto-glutaric Acid | - |
| B3 | D-Melibiose | + | D11 | Rifamycin SV | - | G7 | D-Malic Acid | - |
| B4 | β-Methyl-D-Glucoside | + | D12 | Minocycline | - | G8 | L-Malic Acid | - |
| B5 | Salicin | + | E1 | Gelatin | + | G9 | Bromosuccinic Acid | + |
| B6 | N-Acetyl-D-Glucosamine | + | E2 | Glycyl-L-Proline | + | G10 | Nalidixic acid | - |
| B7 | N-Acetyl-β-D-Mannosamine | + | E3 | D-Alanine | + | G11 | Lithium Chloride | + |
| B8 | N-Acetyl-D-Galactosamine | - | E4 | L-Arginine | -/+ | G12 | Potassium Tellurite | + |
| B9 | N-AcetylNeuraminic acid | - | E5 | L-Aspartic Acid | + | H1 | Tween 40 | + |
| B10 | 1% NaCl | + | E6 | L-Glutamic Acid | + | H2 | γ-Amino-Butyric Acid | + |
| B11 | 4% NaCl | + | E7 | L-Histidine | - | H3 | α-Hydroxy-Butyric Acid | - |
| B12 | 8% NaCl | + | E8 | L-Pyroglutamic Acid | + | H4 | β-Hydroxy-D,L-butyric Acid | - |
| C1 | α-D-Glucose | + | E9 | L-Serine | - | H5 | α-Keto-Butyric Acid | - |
| C2 | α-D-Mannose | + | E10 | Lincomycin | - | H6 | Acetoacetic Acid | + |
| C3 | D-Fructose | + | E11 | Guanidine HCl | - | H7 | Propionic Acid | - |
| C4 | D-Galactose | + | E12 | Niaproof 4 | - | H8 | Acetic Acid | + |
| C5 | 3-Methyl-D-Glucose | - | F1 | Pectin | + | H9 | Formic Acid | -/+ |
| C6 | L-Fucose | - | F2 | Galacturonic acid | + | H10 | Aztreonam | + |
| C7 | D-Fucose | - | F3 | D-Galactonic Acid Lactone | + | H11 | Sodium Butyrate | + |
| C8 | L-Rhamnose | - | F4 | D-Gluconic Acid | -/+ | H12 | Sodium Bromate | -/+ |

**Table S7.** Cell-free culture filtrate from *B. vallismortis* LRB-5 inhibits mycelial growth of plant pathogens.

| Treatment | 1 mL·L^-1^ | 5 mL·L^-1^ | 10 mL·L^-1^ | 15 mL·L^-1^ |
| --- | --- | --- | --- | --- |
| *F. proliferatum* | 24.39±2.39e^1^ | 34.44±0.67g | 54.61±0.28f | 61.33±0.45cd |
| *F. verticillioides* | 39.00±0.33b | 50.06±0.95c | 58.72±1.39e | 62.89±0.89cd |
| *F. oxysporum* | 34.17±0.95c | 43.17±1.17e | 60.11±0.78de | 60.06±16.95cd |
| *F. solani* | 37.22±1.34b | 50.06±0.28c | 52.39±1.17g | 67.11±0.89bcd |
| *Rhizoctonia solani* | 28.22±0.45d | 46.61±1.61d | 61.61±1.06c | 68.89±1.33abc |
| *Alternaria alternata* | 29.72±1.06d | 51.56±0.89c | 54.67±0.34f | 57.33±1.23d |
| *Albifimbria verrucaria* | 39.44±1.23b | 57.39±1.06b | 72.22±0.67b | 78.72±1.61a |
| *Aspergillus flavus* | 16.89±0.45f | 25.22±0.89h | 33.72±0.95h | 73.33±1.56ab |
| *Phytophthora cactorum* | 37.17±2.06b | 41.39±0.72f | 60.94±0.50cd | 65.83±0.39bcd |
| *Phoma macrostoma* | 63.56±0.23a | 68.28±0.84a | 75.78±0.341a | 78.06±0.62a |
| P*enicillium brasilianum* | - | - | - | - |

Note: Row comparison (inhibitory effects of cell-free culture filtrate of LRB-5 on mycelial growth of plant pathogens). Values in columns followed by the same letter are not significantly different according to Duncan's test at *p* < 0.05. Values are mean±standard deviation (*n* = 3). - : Fungal diameter cannot be measured. ^1^Inhibition rate (%) = (D − d) / D × 100%. D = diameter of pathogen growth in control plates (mm); d = diameter of pathogen growth in treated plates (mm).

**Table S8.** The inhibitory effect of volatile organic compounds identified from *B. vallismortis* LRB-5 on the mycelial growth of plant pathogens. Values: means (standard deviation) (n = 3).

| Number | Ingredient name | *F. proliferatum* | *F. verticillioides* | *F. oxysporum* | *F. solani* | *Rhizoctonia solani* | *Alternaria alternata* | *Albifimbria verrucaria* | *Aspergillus flavus* | Penicillium brasilianum | *Phytophthora cactorum* |
| --- | --- | --- | --- | --- | --- | --- | --- | --- | --- | --- | --- |
| A | 5-methyl-2-heptanone | 2.62 (0.02)^1^ | 2.21 (0.01) | 2.52 (0.04) | 2.30 (0.02) | - | 2.61 (0.02) | 2.01 (0.02) | 1.21 (0.02) | - | 1.10 (0.01) |
| B | Heptacosane | 1.83 (0.02) | 1.30 (0.02) | 1.20 (0.02) | 1.62 (0.02) | 1.26 (0.06) | 1.03 (0.02) | - | 0.99 (0.02) | 0.99 (0.02) | 1.61 (0.02) |
| C | Heneicosane | 1.51 (0.01) | 1.49 (0.02) | 1.31 (0.02) | 2.04 (0.03) | 1.30 (0.02) | 1.51 (0.02) | 2.12 (0.03) | 3.02 (0.03) | 1.10 (0.02) | 1.91 (0.02) |
| D | 2,5-Dihydroxybenzaldehyde | 2.01 (0.02) | 2.60 (0.02) | 3.22 (0.02) | 2.60 (0.02) | 0.71 (0.02) | 1.71 (0.02) | - | 1.73 (0.03) | - | 2.37 (0.04) |
| E | 1-Tetradecanol | 2.09 (0.02) | 3.01(0.02) | 2.74 (0.03) | 3.11 (0.03) | 1.11 (0.01) | 2.21 (0.03) | - | 1.10 (0.02) | - | 1.34 (0.01) |
| F | 1-Hexadecanol | 1.99 (0.01) | 1.30 (0.02) | 2.85 (0.03) | 2.62 (0.02) | 0.76 (0.05) | 3.00 (0.02) | - | 1.21 (0.02) | - | 2.15 (0.04) |
| G | 2-Ethyl-1-hexanol | 2.00 (0.02) | 2.02 (0.03) | 2.85 (0.02) | 2.47 (0.03) | - | 3.20 (0.02) | - | 1.41 (0.02) | - | 1.30 (0.01) |
| H | Styrene | 3.50 (0.02) | 2.30 (0.02) | 3.11 (0.04) | 2.64 (0.03) | - | 2.22 (0.02) | - | 1.47 (0.03) | - | 2.13 (0.06) |
| I | Toluene | 2.43 (0.02) | 1.93 (0.02) | 1.94 (0.10) | 1.62 (0.02) | - | 3.03 (0.02) | 1.32 (0.01) | 1.84 (0.04) | - | 3.08 (0.03) |
| Control | Sterile water | + | + | + | + | + | + | + | + | + | + |

Note: - : the fungus was completely suppressed, while + the fungus grows normally, and the inhibitory effect can be ignored.

^1^The diameter of pathogen growth in treated plates (cm).

**Table S9:** Free amino acid contents in extracellular culture medium using HPLC-MS/MS. Values: means (standard deviation of the mean).

| Amino acid | Content (μg^.^mL^-1^) | Standard curve |
| --- | --- | --- |
| Glutamine (Gln) | 21.26 (0.20) | y = 894.92x - 7272.7, *R2* = 0.9998 |
| Asparagine (Asn) | 0.81 (0.00) | y = 40.176x + 580.33, *R^2^* = 0.9998 |
| S-adenosyl methionine (S-Met) | 28.42 (0.36) | y = 547.09x - 5573, *R2* = 0.9996 |
| Citrulline | 0.14 (0.00) | y = 410.38x + 406.91, *R2* = 0.9998 |
| Tryptophan (Trp) | 2.14 (0.00) | y = 3530x - 2009.1, *R2* = 0.9997 |
| L-Kynurenine (Kyn) | 0.16 (0.00) | y = 1422.7x - 4836.2, *R2* = 0.9999 |
| Leucine (Leu) | 14.23 (0.15) | y = 55533x + 2481.9, *R2* = 0.9998 |
| l-isoleucine (Ile) | 0.76 (0.03) | y = 15275x - 1855.1, *R2* = 0.9998 |
| Glycine (Gly) | 0.28 (0.01) | y = 1842.7x + 395.49, *R2* = 0.9998 |
| Alanine (Ala) | 0.66 (0.02) | y = 241.63x + 27.191, *R2* = 0.9996 |
| Serine (Ser) | 2.57 (0.01) | y = 1094x + 150.64, *R2* = 0.9997 |
| Proline (Pro) | 0.33 (0.00) | y = 2339.5x + 178.39, *R2* = 0.9999 |
| Valine (Val) | 31.13 (0.03) | y = 22271x - 1726.2, *R2* = 0.9996 |
| Threonine (Thr) | 3.55 (0.02) | y = 2872.3x + 19.571, *R^2^* = 0.9999 |
| Aspartic acid (Asp) | 4.38 (0.01) | y = 3845.2x + 417.21, *R2* = 0.9999 |
| Lysine (Lys) | 29.91 (0.04) | y = 95831x - 4478.6, *R2* = 1.0000 |
| Glutamate (Glu) | 37.04 (0.01) | y = 32162x - 161.11, *R2* = 0.9997 |
| Phenylalanine (Phe) | 38.75 (0.03) | y = 72563x - 1820, *R2* = 0.9999 |
| Arginine (Arg) | 0.46 (0.01) | y = 151310x + 5809.1, *R2* = 0.9997 |
| Tyrosine (Tyr) | 49.89 (0.02) | y = 49063x + 1045.5, *R2* = 0.9998 |
| Histidine (His) | 6.85 (0.03) | y = 129201x + 4665, *R2* = 0.9998 |
| Methionine (Met) | 41.74 (0.02) | y = 55769x - 4995.6, *R2* = 0.9998 |

Note: Free amino acid content below 0.1 μg^.^mL^-1^ was excluded.

**Table S10:** Effect of different treatments on Margalef Index, McIntosh Index, Brillouin Index, Simpson Index, and Shannon Index based on the T-RFLP data in September. Values in columns followed by the same letter are not significantly different according to Duncan's test at *p* < 0.05 (row comparison). Values are mean±standard deviation (*n* = 3). The Margalef index reflects the abundance of soil microbial communities; the McIntosh index reflects the number of different types of carbon sources utilized, which can be used to distinguish different carbon source utilization levels; and the Brillouin, Simpson, and Shannon indices reflect the diversity of soil microbial communities.

| Microorganism | Treatment | Margalef Index | Shannon Index | Simpson Index | McIntosh Index | Brillouin Index |
| --- | --- | --- | --- | --- | --- | --- |
| Fungi | CK1 | 5.45±0.03b | 2.69±0.04b | 0.82±0.03a | 50.54±0.04a | 1.85±0.05c |
|  | CK2 | 4.34±0.05c | 2.79±0.04a | 0.55±0.05b | 30.68±0.06b | 2.65±0.03b |
|  | T1 | 4.37±0.05b | 2.69±0.06b | 0.82±0.02a | 50.54±0.05a | 1.73±0.02d |
|  | T2 | 5.53±0.05a | 2.81±0.01a | 0.45±0.02c | 30.71±0.01b | 2.85±0.04a |
| Bacterial | CK1 | 4.81±0.03a | 2.93±0.01a | 0.23±0.01c | 20.84±0.01b | 2.77±0.02a |
|  | CK2 | 3.96±0.03d | 2.86±0.05b | 0.34±0.01a | 30.74±0.04a | 2.35±0.03d |
|  | T1 | 4.25±0.04c | 2.92±0.03ab | 0.25±0.04bc | 20.80±0.04b | 2.54±0.02c |
|  | T2 | 4.36±0.02b | 2.90±0.02ab | 0.28±0.01b | 30.79±0.02a | 2.63±0.03b |

**References**

Avery B (1973) Soil classification in the Soil Survey of England and Wales. Journal of Soil Science 24: 324-38.

Chun J, Bae KS (2000) Phylogenetic analysis of *Bacillus subtilis* and related taxa based on partial *gyrA* gene sequences. Antonie Van Leeuwenhoek 78: 123-27.

Dos Santos HRM, Argolo CS, Argôlo-Filho RC, Loguercio LL (2019) A 16S rDNA PCR-based theoretical to actual delta approach on culturable mock communities revealed severe losses of diversity information. BMC microbiology 19: 1-14.

Duan Y, Chen R, Zhang R, Jiang W, Chen X, Yin C, Mao Z (2022) Isolation and identification of *Bacillus vallismortis* HSB-2 and its biocontrol potential against apple replant disease. Biological Control 170: 104921.

Duan YN, Jiang WT, ZhangR, Chen R, Chen XS, Yin CM, Mao ZQ (2022) *Fusarium proliferatum* f.sp. *malus domestica* causing apple replant disease in China. Plant Disease 106: 2958-66.

Fu L, Penton CR, Ruan Y, Shen Z, Xue C, Li R, Shen Q (2017) Inducing the rhizosphere microbiome by biofertilizer application to suppress banana Fusarium wilt disease. Soil Biology and Biochemistry 104: 39-48.

Peverill K, Sparrow L, Reuter D (1999) Soil Analysis: An Interpretation Manual CSIRO. Collingwood, Australia: 170-74.

Quéric NV, Soltwedel T (2012) Benthic prokaryotic community dynamics along the Ardencaple Canyon, Western Greenland Sea. Sedimentary Geology 263: 36-44.

Xiang L, Wang M, Jiang W, Wang Y, Chen X, Yin C, Mao Z (2021) Key indicators for renewal and reconstruction of perennial trees soil: Microorganisms and phloridzin. Ecotoxicology and Environmental Safety 225: 112723.

Yamamoto S, Harayama S (1995) PCR amplification and direct sequencing of *gyrB* genes with universal primers and their application to the detection and taxonomic analysis of *Pseudomonas putida* strains. Applied and environmental microbiology 61: 1104-09.

Zalila-Kolsi I, Mahmoud AB, Ali H, Sellami S, Nasfi Z, Tounsi S, Jamoussi K (2016) Antagonist effects of *Bacillus* spp. strains against *Fusarium graminearum* for protection of durum wheat (*Triticum turgidum* L. subsp. *durum*). Microbiological research 192: 148-58.
